# Apoptotic Vesicles Derived from Mesenchymal Stem Cells Ameliorate Hypersensitivity Responses via Inducing CD8^+^ T Cells Apoptosis with Calcium Overload and Mitochondrial Dysfunction

**DOI:** 10.1002/advs.202407446

**Published:** 2025-03-16

**Authors:** Anqi Liu, Peng Peng, Changze Wei, Fanhui Meng, Xiaoyao Huang, Peisheng Liu, Siyuan Fan, Xinyue Cai, Meiling Wu, Zilin Xuan, Qing Liu, Xinyu Qiu, Zhenlai Zhu, Hao Guo

**Affiliations:** ^1^ State Key Laboratory of Oral & Maxillofacial Reconstruction and Regeneration National Clinical Research Center for Oral Diseases Shaanxi Clinical Research Center for Oral Disease Department of Preventive Dentistry School of Stomatology The Fourth Military Medical University Xi'an Shaanxi 710032 China; ^2^ Department of Stomatology 985 Hospital of Joint Logistics Support Force Taiyuan Shanxi 030000 China; ^3^ Department of Chemical and Biomolecular Engineering National University of Singapore 4 Engineering Drive 4 Singapore 117585 Singapore; ^4^ Faculty of Medicine and Health University of Sydney Camperdown NSW 2050 Australia; ^5^ State Key Laboratory of Oral & Maxillofacial Reconstruction and Regeneration National Clinical Research Center for Oral Diseases Shaanxi Clinical Research Center for Oral Disease Department of Oral Medicine School of Stomatology The Fourth Military Medical University Xi'an Shaanxi 710032 China

**Keywords:** apoptotic vesicles, CD8^+^ T cells, mesenchymal stem/stromal cells, mitochondrial dysfunction, type IV hypersensitivity responses

## Abstract

Apoptosis is crucial for maintaining internal homeostasis. Apoptotic vesicles (ApoVs) derived from mesenchymal stem/ stromal cells (MSCs‐ApoVs) as natural lipid nanoparticles are attractive candidates for the next generation of immunotherapies. However, the therapeutic potential of MSCs‐ApoVs in managing hypersensitivity reactions mediated by CD8^+^ T cells remains elusive. This research utilized contact hypersensitivity and oral lichenoid reaction models, both of which represent type IV hypersensitivity reactions. ApoVs are shown that derived from stem cells from human exfoliated deciduous teeth (SHED‐ApoVs), a subtype of MSCs, directly fused with the plasma membrane of CD8^+^ T cells, subsequently increasing membrane permeability through L‐type voltage‐gated Ca^2+^ channels. This initiates a cascade of events including calcium overload, mitochondrial dysfunction, and the initiation of apoptosis in these cells. As known, this is the first study to characterize SHED‐ApoVs as immune microenvironment modulators, demonstrating their therapeutic potential and mechanism in these reactions. Moreover, analysis of blood samples from patients with oral lichenoid reactions verified the antihypersensitivity property of SHED‐ApoVs. This study sheds light on the therapeutic prospects of MSCs‐ApoVs and their underlying mechanisms in diseases mediated by CD8^+^ T cells, contributing novel perspectives for the clinical application of ApoVs and nanovesicle‐based cell‐free therapies.

## Graphical abstract

The therapeutic potential of ApoVs derived from stem cells from human exfoliated deciduous teeth (SHED‐ApoVs) in the treatment of CD8^+^ T cell‐mediated hypersensitivity reactions is noteworthy. SHED‐ApoVs are capable of fusing with the plasma membrane of CD8^+^ T cells, which subsequently triggers a series of events characterized by calcium overload, mitochondrial dysfunction, and the initiation of apoptosis in these cells.

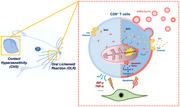

## 1 Introduction

Apoptosis serves as the predominant form of programmed cell death, essential for preserving internal homeostasis.^[^ ^1^ ^]^ Apoptotic vesicles (ApoVs) are natural lipid nanoparticles that encapsulate apoptotic cell components during biogenesis, inheriting diverse and unique properties from their parent cells.^[^ ^2^ ^]^ However, the precise therapeutic role of ApoVs derived from various cell sources remains elusive. To date, numerous patients have undergone transplantation with culture‐expanded mesenchymal stem/ stromal cells (MSCs) for the treatment of various immune diseases, including systemic lupus erythematosus and rheumatoid arthritis.^[^ ^3^ ^]^ Mounting evidences indicate that the therapeutic efficacy of MSCs transplantation may be associated with the secretion of MSC‐derived ApoVs (MSC‐ApoVs). This is because transplanted MSCs are swiftly recognized and eliminated by the host immune system, while still retaining their immunoregulatory function.^[^ ^4^ ^]^ Compared to the hurdles faced in MSC therapy, such as ethical concerns and tumor formation, MSCs‐ApoVs offer a promising alternative. Their tissue repair‐promoting characteristics and immunoregulatory potential, inherited from their parent cells, make these natural lipid nanoparticles attractive candidates for advancing nanovesicle‐based cell‐free immunotherapies.

Stem cells from human exfoliated deciduous teeth (SHED), a subtype of MSCs originating from the early neural crest stem cells, possess optimal immunomodulatory and regenerative properties.^[^ ^5^ ^]^ Studies have demonstrated that SHED exhibits a more favorable cytokine profile in relation to inflammatory, immunomodulatory, proliferative, and osteogenic potential compared to human dental pulp stem cells (hDPSCs).^[^ ^6^ ^]^ Moreover, SHED displays significant effects in inhibiting T helper 17 cells when compared to human bone marrow mesenchymal stem cells (hBMSCs).^[^ ^7^ ^]^ Previously, our team confirmed the ideal anti‐inflammatory and regenerative properties of SHED and successfully achieved tooth regeneration in both animal models and clinical trials.^[^ ^5^, ^8^ ^]^ Additionally, it has been reported that the immunomodulatory potential of SHED in ameliorating gland inflammation and dryness symptoms of Sjögren's syndrome via secreting soluble programmed cell death ligand 1 (PD‐L1).^[^ ^9^ ^]^ Furthermore, we verified that exosomes and ApoVs derived from SHED serve as modulators of the inflammatory microenvironment, thereby promoting the angiogenesis process during the regeneration of dental pulp tissue.^[^ ^10^ ^]^ However, whether SHED‐derived ApoVs (SHED‐ApoVs) are regulators of the immune microenvironment, possessing immunomodulatory function as their parent cells remains largely unclear. Investigating the immunomodulatory properties of SHED‐ApoVs and elucidating their impact on immune cells has the potential to broaden their clinical application and underscore their promise as immune therapeutics.

The cytotoxic function of CD8^+^ T cells against tumor cells plays a crucial role in immune surveillance against cancer. Nonetheless, when autoreactive responses occur and target the body's own cells, CD8^+^ T cells can result in autoimmune disorders, graft rejection reactions, and type IV hypersensitivity reactions.^[^ ^11^ ^]^ Type IV hypersensitivity is involved in the pathogenesis of various diseases, including allergic contact dermatitis, oral lichenoid reactions (OLR), and multiple sclerosis.^[^ ^12^ ^]^ Given the intricate etiology and varied manifestations of type IV hypersensitivity, the primary treatment focus revolves around symptom management. Factors like medication effectiveness, optimal glucocorticoid timing, and patient tolerability are key determinants of treatment outcome.^[^ ^13^ ^]^ Thus, the advancement of tailored and potent immunotherapy strategies is essential in this research field. Type IV hypersensitivity, commonly referred to as delayed hypersensitivity, typically manifests 24–72 h after exposure and is distinguished by two distinct phases. The sensitization phase involves events following the initial exposure to the hapten, leading to the development of CD8^+^ T cells. During the elicitation phase, re‐exposure of sensitized individuals to the hapten results in clinical manifestations.^[^ ^14^ ^]^ However, there is still insufficient knowledge regarding the therapeutic potential of ApoVs on hypersensitivity responses. Exploring this area will provide new insights into the physiology of ApoVs and offer a promising approach for the management of CD8^+^ T cell‐related diseases, such as viral infections, tumors, and autoimmune diseases.

**Scheme 1:**
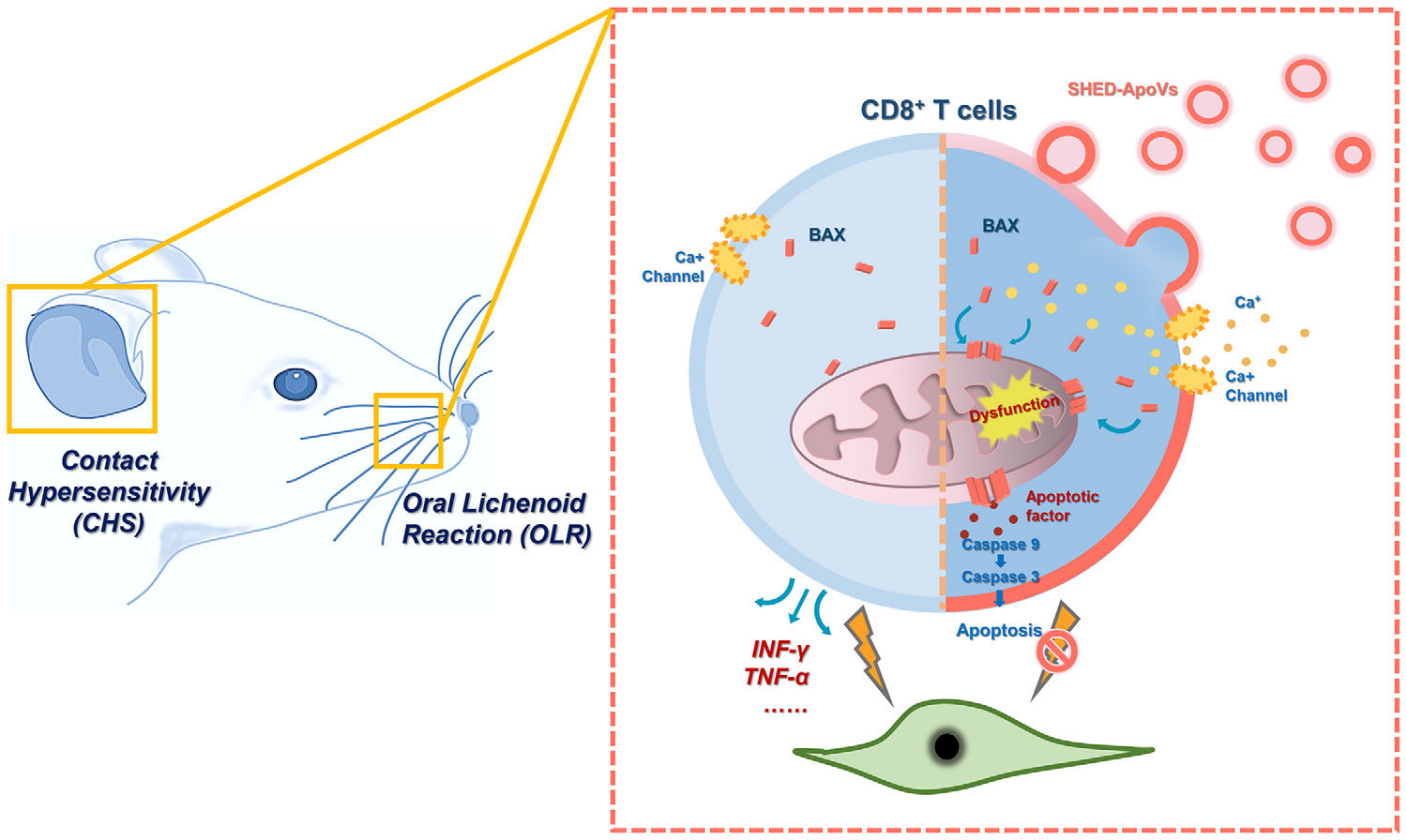
The schematic diagram demonstrates the ApoVs interact with CD8^+^ T cells via membrane fusion, triggering cascade reactions including calcium overload, mitochondrial dysfunction, BAX translocation, and eventual apoptosis in CD8^+^ T cells.

This research aims to explore the potential of ApoVs as nano‐mediators in the immune microenvironment for modulating type IV hypersensitivity. We characterized SHED‐ApoVs and employed contact hypersensitivity (CHS) and oral lichenoid reactions (OLR) models, both of which represent type IV hypersensitivity reactions, to investigate the influence of ApoVs on CD8^+^ T cells. Interestingly, we uncovered the therapeutic role of ApoVs in type IV hypersensitivity reactions and first revealed a significant anti‐hypersensitivity function of SHED‐ApoVs on CD8^+^ T cells, which was confirmed by patients with type IV hypersensitivity. Mechanically, ApoVs interact with CD8^+^ T cells via membrane fusion, resulting in upregulation of L‐type voltage‐gated Ca^2+^ channels. This triggers a cascade of reactions including calcium overload, mitochondrial dysfunction, BAX translocation, and eventual apoptosis in CD8^+^ T cells (**Scheme** **1**). The findings provide a foundation for further preclinical research on MSCs‐ApoVs and endorse the application of nanovesicle‐based cell‐free therapy for diseases mediated by CD8^+^ T cells.

## 2 Results

### 2.1 Isolation and Characterization of MSCs and MSCs‐ApoVs

SHED as a kind of MSCs was used in this study, which possessed general MSCs characteristics including the capacity for self‐renewal and multipotent differentiation (Figure S1A, Supporting Information). Furthermore, these cells expressed MSCs surface molecules including CD90, CD105, CD73, and CD29, while showing negative expression of leukocyte and hematopoietic progenitor cell surface markers CD14, HLA‐DR, CD45, CD34, and CD79a (Figure S1B, Supporting Information). Apoptosis in SHED was induced using Staurosporine (STS) for 16 h, and ApoVs were isolated through an established sequential certification process (Figure S1C, Supporting Information). The characteristic morphology changes, such as swelling and spherification in apoptosis of SHED were observed by microscope (Figure S1D, Supporting Information). Flow cytometry analysis confirmed an increased percentage of apoptotic SHED using Annexin V/PI double staining (Figure S1E, Supporting Information). Transmission electron microscopy (TEM) demonstrated that ApoVs displayed a classical double‐membrane spherical structure (Figure S1F, Supporting Information). Nanoparticle tracking analysis (NTA) indicated that the size of ApoVs ranged from 400 to 600 nm (Figure S1G, Supporting Information). ApoVs expressed common extracellular vesicles (EVs) markers CD63, CD9, and TSG101, but failed to express exosomes‐specific marker GM130. Compared to uninduced SHED, ApoVs expressed the specific apoptosis‐associated marker cleaved‐caspase3 (Figure S1H, Supporting Information). These findings verified that the ApoVs derived from SHED in this study exhibit the specific characteristics of high‐purity apoptotic vesicles originating from MSCs.

### 2.2 MSCs‐ApoVs Treatment Attenuated CD8^+^ T Cells‐mediated Contact Hypersensitivity

**Figure 1:**
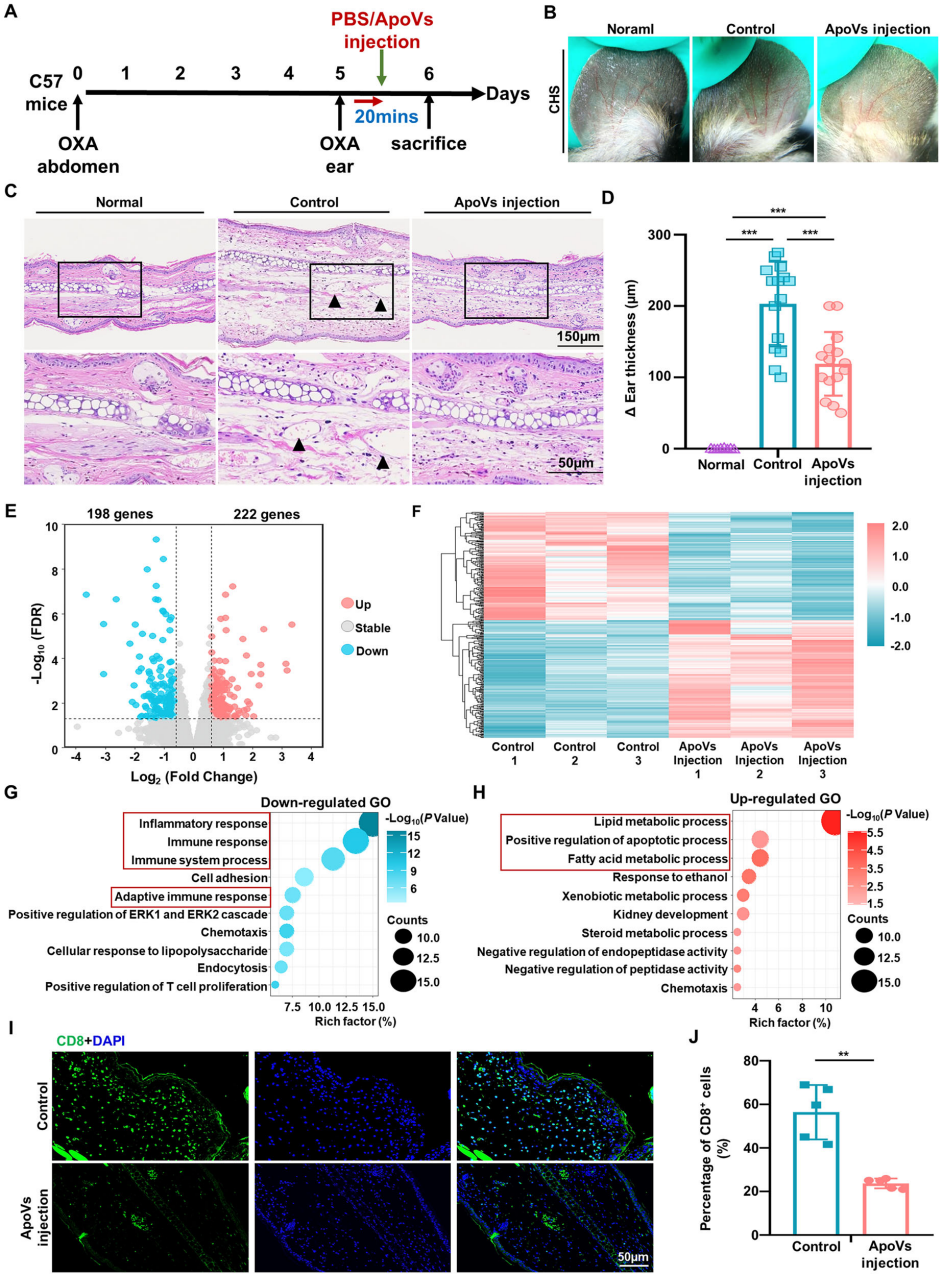
MSCs‐ApoVs Treatment Attenuated CD8^+^ T Cells‐mediated Contact Hypersensitivity. A) Schematic illustration of contact hypersensitivity experimental design. B) Representative phenotype of ears captured by dermoscopy. C) Hematoxylin‐eosin (H&E) staining of ear samples collected 24 h after the challenge. The lower panel magnifies the boxed area in the top panel. Scale bar: 150 µm for the upper panel and 50 µm for the lower panel. Black arrowheads indicate dilated capillaries. D) Ear thickness was measured 24 h after the elicitation in three groups (*n* = 15). E) Volcano plots displayed the profile of differentially expressed genes of ear samples collected form the Control and ApoVs injection groups (*n* = 3). The 222 upregulated DEGs (red) and 198 downregulated DEGs (blue) were shown in plots. F) Heatmap exhibited the profiles of upregulated and downregulated DEGs in the ApoVs injection group compared to the Control group (*n* = 3). G‐H) Gene Ontology (GO) of biological processes enrichment analysis related to the downregulated and upregulated DEGs in ear samples. The top 10 enriched terms were presented as bubble charts. I‐J) Representative images and quantification of CD8^+^ T cells in the Control and ApoVs injection groups (*n* = 5). Scale bar: 50 µm. ** *p *< 0.01, ***p < 0.001.

In this study, a mouse CHS model was established using oxazolone (OXA) as a hapten, following a previously described protocol.^[^ ^14^, ^15^ ^]^ Briefly, mice were sensitized epicutaneously on the shaved abdomen with 100 µL of 3.0% OXA on day 0. Five days later, OXA was challenged on both ears. After 20 min of OXA challenge, ApoVs were intradermally injected into one ear while the other ear served as the control group which was injected with phosphate‐buffered saline (PBS) (**Figure** **1**A). The varying degree of swelling between the left and right ears showed that ApoVs significantly inhibited ear swelling in a dose‐dependent manner compared to the control group. A concentration of 20 mg mL^−1^ ApoVs was selected for subsequent in vivo studies (Figure S2A, Supporting Information). To confirm the presence of ApoVs at the lesion site, the membrane of ApoVs was labeled with PKH26. Following injection of labeled ApoVs into the lesioned ears of mice, the ears were collected after 6 h and examined via immunofluorescence staining. Immunofluorescence staining revealed the PKH26‐positive cells within the lesion area, indicating successful injection of ApoVs (Figure S2B, Supporting Information). Dermoscopic analysis revealed that the control group displayed elicited reactions such as slight local skin hyperemia and redness compared with the normal group, while the ApoVs injection group showed attenuated reactions (Figure 1B). Hematoxylin and eosin (H&E) staining revealed the presence of spongiosis and capillary dilation in the control group, which differed from the observed characteristics in both normal ears and the group that received ApoVs injection (Figure 1C). Ear thickness measurements using an Engineer's micrometer caliper before and after the challenge showed a notable suppression of ear swelling in the ApoVs injection group (Figure 1D; Figure S2C, Supporting Information).

To identify the gene expression profile of ear tissue treated with ApoVs, RNA transcriptomic sequencing was performed on ear samples from the control and ApoVs injection group. Volcano maps illustrated the magnitude of fold change for all genes with false discovery rate (FDR), in the ApoVs injection group compared to the control group. The profiles of 222 differentially upregulated and 198 downregulated genes (DEGs) were shown in volcano plots and heatmap (Figure 1E,F). Moreover, Gene ontology (GO) analysis of biological processes exhibited the top 10 gene enrichment pathways for upregulated and downregulated DEGs. The downregulated genes were notably associated with inflammatory and immune response, implying that ear hypersensitivity leads to tissue inflammatory and immune response. The upregulated genes were primarily linked to metabolic and apoptotic processes, implying that ApoVs treatment accelerated local tissue metabolic and apoptotic processes (Figure 1G,H). qRT‐PCR analysis of ear tissues after ApoVs treatment confirmed a decreased level of inflammation, with lower expression of Ifng (IFN‐γ), Il1b (IL‐1β), and Il6 (IL‐6), and reduced expression of T cell‐attracting chemokines like Cxcl9, Cxcl10, and Cxcl11 (Figure S2D,E, Supporting Information). Immunostaining of endothelial markers CD31 in ear lesions displayed a decrease of capillary dilation in the ApoVs injection group, consistent with H&E finding (Figure S2F,G, Supporting Information). Simultaneously, following ApoVs treatment, immunostaining showed decreased expression of pro‐inflammatory TNF‐α^+^ cells and increased anti‐inflammatory TGF‐β^+^ cells in the lesion site compared to the control group (Figure S2H,I, Supporting Information). In addition, there was a reduction in the percentage of CD8^+^ T cells at lesional sites after 24 h of ApoVs injection (Figure 1I,J). The data presented implicate that treatment with SHED‐ApoVs effectively ameliorates hypersensitivity‐induced inflammatory and immune responses by potentially decreasing the percentage of CD8^+^ T cells in the affected area.

### 2.3 ApoVs‐afforded Antihypersensitivity Effects by Promoting the Apoptosis of CD8^+^ T Cells

**Figure 2:**
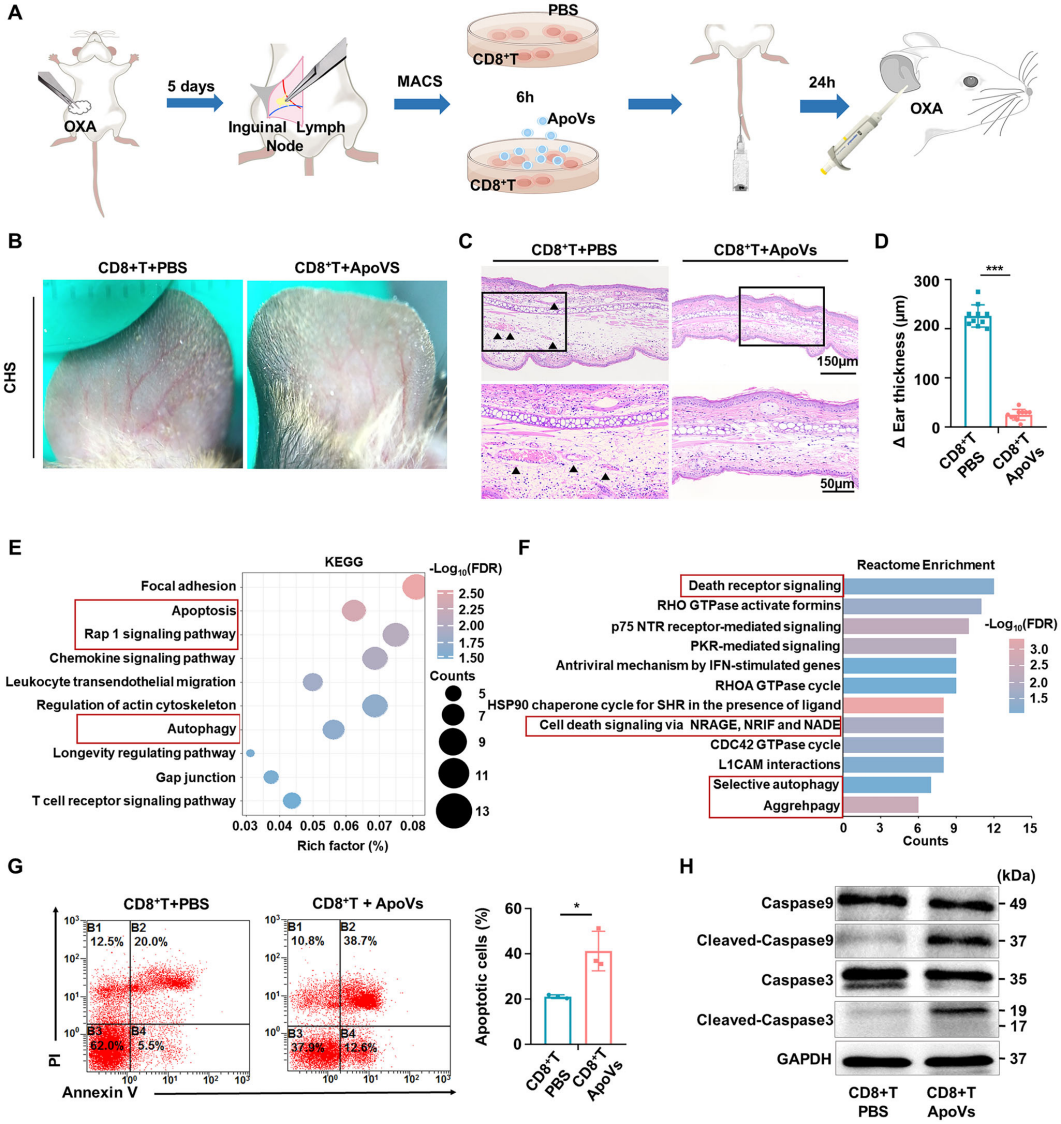
ApoVs‐afforded Anti‐hypersensitivity Effects by Promoting the Apoptosis of CD8^+^ T Cells. A) Schemes of the adoptive transfer experimental design. CD8^+^ T cells from the draining lymph nodes of oxazolone‐sensitized WT mice were isolated and cultured with or without ApoVs. Naïve WT mice then received adoptive transfer of these CD8^+^ T cells, followed by treatment on the ears of recipient mice with OXA 2 h post‐transfer. B) Representative ear lesions visualized by dermoscopy. C) H&E staining of ear samples collected 24 h after the challenge. The lower panel magnifies the boxed area in the top panel. Scale bar: 150 µm for the upper panel and 50 µm for the lower panel. Black arrowheads indicate dilated capillaries. D) Ear thickness was measured 24 h after the elicitation in groups receiving intravenous infusion of CD8^+^ T cells treated with PBS or ApoVs (*n* = 10). E) Bubble diagram displayed the top 10 KEGG pathways enriched terms of upregulated DEGs in ApoVs‐treated CD8^+^ T cells compared to PBS‐treated CD8^+^ T cells (*n* = 3). F) The bar chart showed the top 12 Reactome enrichment pathways of upregulated DEGs in ApoVs‐treated CD8^+^ T cells compared to PBS‐treated CD8^+^ T cells (*n* = 3). G) Annexin V/PI double staining showed that ApoVs induced CD8^+^ T cell apoptosis (*n* = 3). H) Westen blot displayed the expression level of classical apoptosis protein cleaved‐Caspase 9 and cleaved‐Caspase 3 in CD8^+^ T cells after ApoVs treatment. * *p *< 0.05, ***p < 0.001, MACS: Magnetic‐Activated Cell Sorting.

Next, to validate whether ApoVs alleviated hypersensitivity reactions by specifically targeting CD8^+^ T cells during the elicitation phase, an adoptive transfer experiment was carried out. Specifically, cells were collected from the inguinal lymph nodes of OXA‐sensitized mice 5 days after sensitization and then enriched for sensitized CD8^+^ T cells. The sorted sensitized CD8^+^ T cells were treated with PBS or ApoVs and adoptively transferred to unsensitized mice via tail vein injection (**Figure** **2**A). Flow cytometric analysis confirmed the purity of sorted CD8^+^ T cells (CD3^+^ CD8^+^ T cells) (Figure S3A, Supporting Information). The optimal concentration of ApoVs (10 µg/mL) was determined based on in vitro analysis of IFN‐γ production, a marker of CD8^+^ T cell function (Figure S3B, Supporting Information). Subsequent ELISA analysis revealed a decrease in the expression of CD8^+^ T cells effector cytokines, including IFN‐γ, TNF‐α, perforin‐1, and granzyme B, after treatment with ApoVs (Figure S3C–F, Supporting Information). Following exposure to OXA, the ear response of CD8^+^ T cells treated with PBS displayed signs of redness and hyperemia, indicating the success of the adoptive transfer experiment. Furthermore, H&E staining and micrometer caliper measurements showed the recipient mice injected with ApoVs‐treated CD8^+^ T cells had significantly decreased ear redness and swelling compared to those injected with PBS‐treated CD8^+^ T cells (Figure 2B–D). Additionally, IHC staining of ear lesions revealed a significant reduction in enlarged blood vessels and the presence of pro‐inflammatory TNF‐α positive cells following treatment with ApoVs (Figure S4A,B, Supporting Information). In this study, it was observed that the ear hypersensitivity response in recipient mice was dependent on the sensitized CD8^+^ T cells that were injected. These findings imply a direct inhibitory effect of ApoVs on CD8^+^ T cells during the elicitation phase of hypersensitivity reactions.

To further understand the mechanisms underlying the effects of ApoVs on CD8^+^ T cells, RNA transcriptomic sequencing was performed on ApoVs‐treated CD8^+^ T cells, with PBS‐treated CD8^+^ T cells as a control. Kyoto Encyclopedia of Genes and Genomes (KEGG) pathway analysis exhibited that the upregulated genes in the ApoVs‐treated group were significantly enriched in terms related to apoptosis‐relative signaling (Figure 2E). Similarly, Reactome pathway enrichment analysis showed that the upregulated genes were enriched in terms involving cell death‐related signaling (Figure 2F). Hence, we examined the apoptosis levels of CD8^+^ T cells subsequent to ApoVs administration. Flow cytometric analysis revealed an increase in the percentage of Annexin V‐positive apoptotic CD8^+^ T cells following ApoVs treatment (Figure 2G). ApoVs treatment markedly induced CD8^+^ T cells apoptosis with upregulated expression of specific apoptosis‐associated markers cleaved caspase‐9 and cleaved caspase‐3 (Figure 2H). The co‐localization of apoptotic marker TUNEL and CD8^+^ T cells at lesion sites 6 h post‐ApoVs injection further confirmed the apoptosis of CD8^+^ T cells after ApoVs treatment in vivo (Figure S4C, Supporting Information). The findings revealed that ApoVs not only repressed the function of CD8^+^ T cells in vivo but also triggered their apoptosis. The presented findings indicate that treatment with ApoVs notably ameliorated hypersensitivity responses via attenuating the function and inducing apoptosis of CD8^+^ T cells.

### 2.4 ApoVs Induced Apoptosis in CD8^+^ T cells via Affecting Mitochondrial Morphology and Function

**Figure 3:**
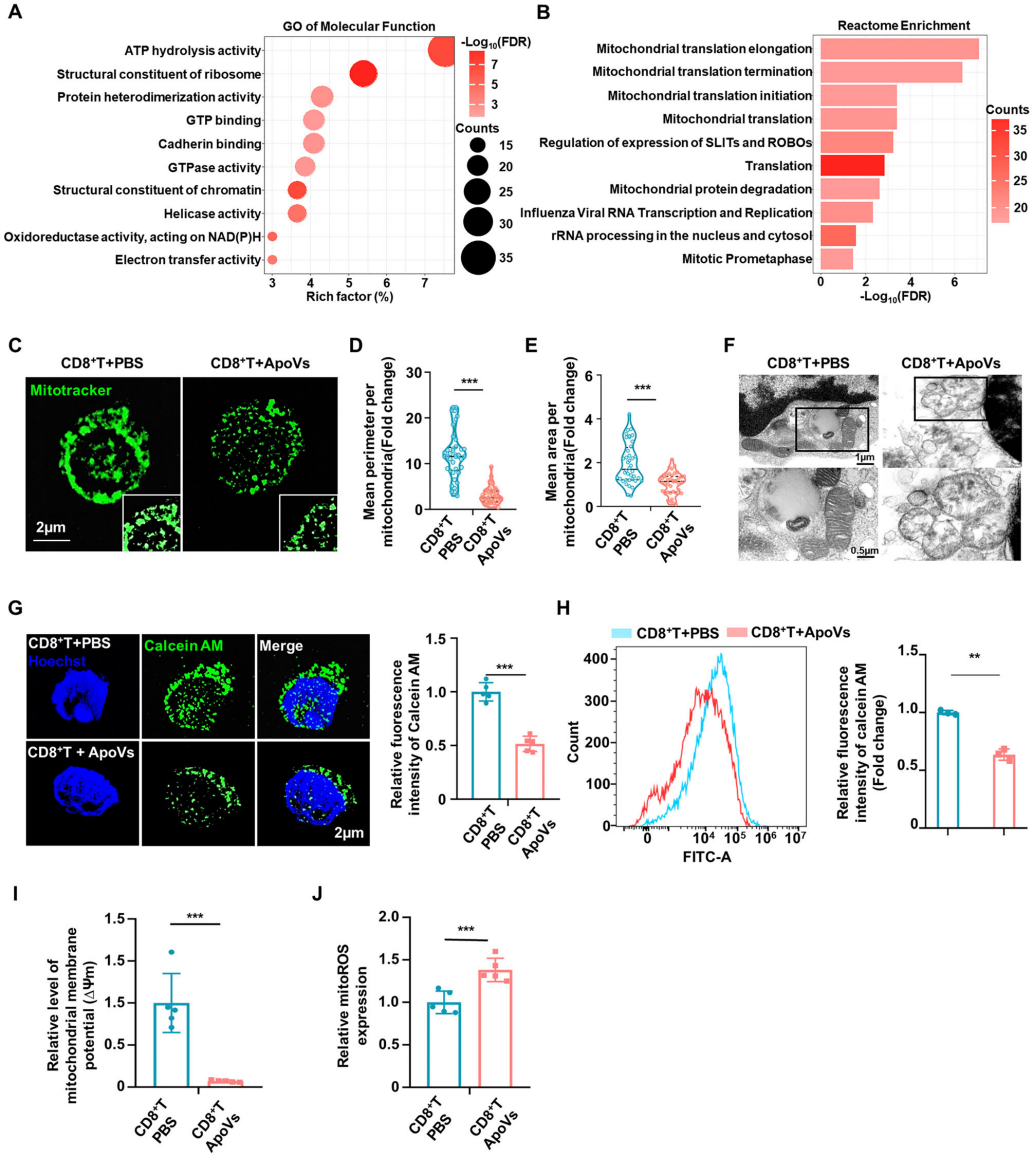
ApoVs Induced Apoptosis in CD8^+^ T cells via Affecting Mitochondrial Morphology and Function. A) Proteomic analysis of ApoVs comparing with EVs showed that GO top 10 enrichment terms of upregulated DEPs, categorized into “Molecular Function” (*n* = 3). B) The bar chart showed the top 10 Reactome enrichment pathways of upregulated DEPs in ApoVs, compared to EVs (*n* = 3). C) Doubling the resolution of structured illumination microscopy (SIM^2^) showed the fragmentation of mitochondrial morphology in CD8^+^ T cells after ApoVs treatment. Scale bar: 2 µm. D‐E) The mean perimeter and mean area of mitochondria in ApoVs treated CD8^+^ T cells significantly decreased (*n* = 40). F) Transmission electron microscopy images revealed the mitochondrial morphology. The lower panel magnifies the boxed area in the top panel. Scale bar: 1 µm for the upper panel and 0.5 µm for the lower panel. G) SIM^2^ exhibited the mitochondrial permeability increased in ApoVs treated CD8^+^ T group, represented by decreasing of relative fluorescence intensity of Calcein AM (*n* = 5). Scale bar: 2 µm. H) Flow cytometry analysis exhibited the relative fluorescence intensity of Calcein AM (*n* = 3). I) Mitochondrial membrane potential (∆Ψm) was analyzed by the relative ration of JC‐1 aggregates (OD = 525) and monomer (OD = 490) (*n* = 5). J) Relative mitochondrial ROS level of two groups (*n* = 5). ** *p *< 0.01, ***p < 0.001.

To delve deeper into the potential molecular mechanisms of ApoVs, proteomic analysis of ApoVs was performed. Considering that ApoVs represent a specific subtype of extracellular vesicles (EVs) released by cells under particular cellular conditions, we utilized EVs originating from the same cellular source as the control group to ensure comparability. GO of molecular function exhibited the top 10 enrichment pathways of the upregulated proteins significantly enriched in terms related to mitochondrial function, such as ATP hydrolysis activity and electron transfer activity (**Figure** **3**A). Similarly, Reactome pathway enrichment analysis showed enrichment of upregulated proteins in mitochondrial‐related terms, including mitochondrial translation termination and mitochondrial protein degradation (Figure 3B). The enrichment analysis consistently pointed toward mitochondrial‐related pathways, implying ApoVs harness CD8^+^ T cells apoptosis via affecting mitochondrial pathways.

Thus, we used the MitoTracker kit to trace mitochondrial morphology. Lattice doubling the resolution of structured illumination microscopy (SIM^2^) revealed that ApoVs treatment resulted in a distinct alteration in the mitochondrial structure of CD8^+^ T cells, characterized by punctate and fragmented morphology. This was accompanied by a significant reduction in both the average perimeter and area of individual mitochondria. (Figure 3C–E). TEM further showed changes in mitochondrial shape from tubular to spherical with translucent morphology following ApoVs treatment (Figure 3F). Furthermore, a mitochondrial permeability transition pore (MPTP) assay kit was employed to assess mitochondrial permeability. A decrease in fluorescence intensity of Calcein AM was indicative of increased permeability of the mitochondria. Both SIM^2^ and flow cytometric analysis revealed a reduction in Calcein AM fluorescence intensity in CD8^+^ T cells following ApoVs treatment, indicating that ApoVs treatment results in heightened permeability of mitochondria in CD8^+^ T cells (Figure 3G,H). Then, a mitochondrial membrane potential assay kit with JC‐1 was applied. The data revealed a dramatic decline of mitochondrial membrane potential in CD8^+^ T cells after ApoVs treatment, implying the depolarization of the mitochondrial membrane potential and early onset of apoptosis of CD8^+^ T cells subsequent to ApoVs treatment (Figure 3I). Moreover, the assessment of mitochondrial reactive oxygen species (ROS) levels indicated a rise in mitoROS accumulation in CD8^+^ T cells exposed to ApoVs (Figure 3J). In this study, the findings demonstrated that treatment with ApoVs triggered apoptosis in CD8^+^ T cells by affecting the morphology and function of mitochondria.

### 2.5 ApoVs Evoked Calcium Influx through Membrane Fusion with CD8^+^ T Cells

**Figure 4:**
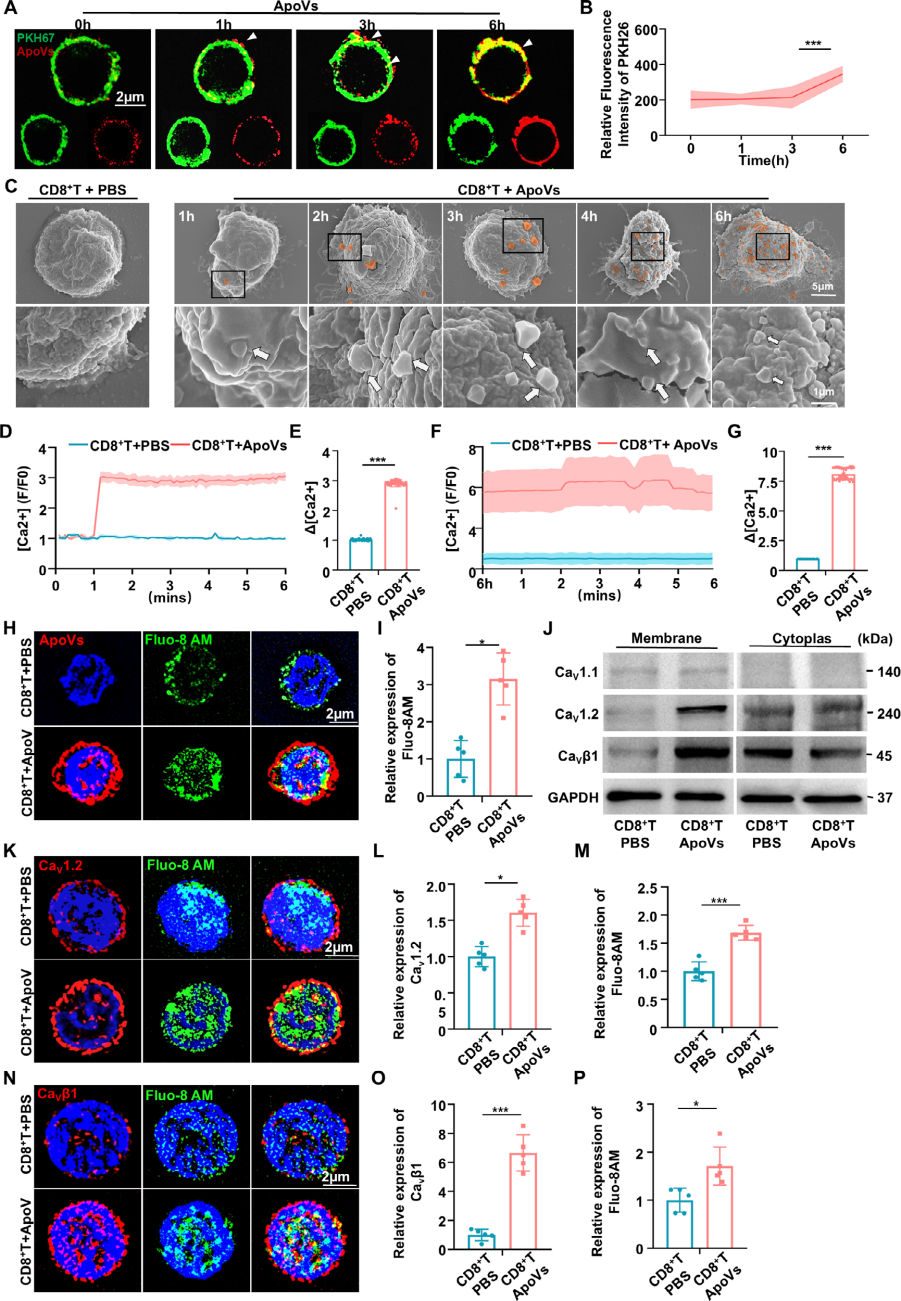
ApoVs Evoked Calcium Influx through Membrane Fusion with CD8^+^ T Cells. A) SIM^2^ showed the ApoVs (red) fused with the membrane of CD8^+^ T cells (green) in a time manner. Scale bar: 2 µm. B) Relative fluorescence intensity of PKH26 (ApoVs) enhanced after 6 h in CD8^+^ T cells treated with ApoVs (*n* = 3). C) SEM showed a sequential observation of ApoVs contact with CD8^+^ T cells. D) Cytosolic Ca^2+^ levels in CD8^+^ T cells after being treated with ApoVs or PBS in 6 min (*n* = 5). E) Quantification of the mean maximal [Ca^2+^] rises (Δ[Ca^2+^]) in CD8^+^ T cells when treated with ApoVs or PBS in 6 min (*n* = 60). F) Cytosolic Ca^2+^ levels in CD8^+^ T cells treated with ApoVs or PBS after 6 h (*n* = 5). G) Quantification of the mean maximal [Ca^2+^] rises (Δ[Ca^2+^]) in CD8^+^ T cells after being treated with ApoVs or PBS after 6 h (*n* = 47). H) SIM^2^ displayed the ApoVs (red) promote calcium probe Fluo‐8 AM (green) expression in CD8^+^ T cells. Scale bar: 2 µm. I) Quantification of the relative expression of Fluo‐8 AM (*n* = 5). J) Western blot showed the expression level of L‐type voltage‐gated calcium channel protein in the membrane and cytoplasm respectively. K) SIM^2^ showed the Ca_v_1.2 (red) and calcium probe Fluo‐8 AM (green) expression in CD8^+^ T cells. Scale bar: 2 µm. L‐M) Quantification of the relative expression of Ca_v_1.2 and Fluo‐8 AM (*n* = 5). N) SIM^2^ showed the Cavβ1 (red) and calcium probe Fluo‐8 AM (green) expression in CD8^+^ T cells. Scale bar: 2 µm. O‐P) Quantification of the relative expression of Ca_v_β1 and Fluo‐8 AM (*n* = 5). * *p *< 0.05, ***p < 0.001.

To further investigate the specific molecular mechanism of how ApoVs induce apoptosis of CD8^+^ T cells, a time‐lapse study was designed to observe the interaction pattern between ApoVs and CD8^+^ T cells. The membrane of CD8^+^ T cells was labeled with green PKH67, while the ApoVs membrane was labeled with red PKH26. Strikingly, SIM^2^ imaging demonstrated a time‐dependent fusion of ApoVs membranes with CD8^+^ T cell membranes, with a significant increase in red PKH26 fluorescence intensity on CD8^+^ T cells observed after 6 h of ApoVs exposure. This time point was selected for further investigation post‐ApoVs treatment (**Figure** **4**A,B). Furthermore, co‐culture experiments involving ApoVs and CD8^+^ T cells were conducted, demonstrating a time‐dependent accumulation of ApoVs (red) on the membrane of CD8^+^ T cells (green) over a 7‐hour period (Figure S5A,B, Supporting Information). To determine the percentages of ApoVs that would fuse with CD8^+^ T cells membrane, the ratio of intracellular and cytomembrane fluorescence intensity of PKH26 (ApoVs) were counted. The finding showed that the fluorescence intensity of intracellular significantly increased after 6 h, implying the membrane of ApoVs was gradually devoured by CD8^+^ T cells after 6 h (Figure S5C, Supporting Information). To further investigate the interaction between ApoVs and CD8^+^ T cells, we conducted a sequential observation of CD8^+^ T cells after ApoVs treatment using scanning electron microscopy (SEM). The data showed that the presence of ApoVs on the CD8^+^ T cell membrane increased in a time‐dependent manner. Remarkable membrane fusion was observed 4 h after ApoVs treatment (Figure 4C). Collectively, the data presented indicate that SHED‐ApoVs induced apoptosis in CD8^+^ T cells mainly via membrane fusion, leading to mitochondria dysfunction.

Considering the impact of calcium on modulating mitochondrial function, apoptotic signaling, as well as T cells activity, we hypothesized that membrane fusion with CD8^+^ T cells triggers calcium influx, ultimately leading to cell apoptosis. Fluo‐8‐AM calcium influx assay was performed, revealing that ApoVs treatment instantaneously increased intracellular calcium ions in CD8^+^ T cells, persisting for up to 6 h. This suggests that ApoVs induced a robust and sustained elevation of calcium levels in CD8^+^ T cells (Figure 4D–G). SIM^2^ further confirmed the heightened expression of calcium ions in CD8^+^ T cells following 6 h of ApoVs treatment (Figure 4H,I). Then, we applied Western blot to examine the levels of membrane and cytoplasmic L‐type Ca^2+^ channel proteins respectively, including Ca_v_1.1, Ca_v_1.2, and regulatory Ca_v_β1. The finding showed that ApoVs treatment enhanced the membrane expression of Ca_v_1.2 and Ca_v_β1, while no significant change was observed in Ca_v_1.1 (Figure 4J). Subsequent immunofluorescence staining displayed that the expression levels of Ca_v_1.2 and Ca_v_β1 increased with calcium influx in CD8^+^ T cells after ApoVs treatment (Figure 4K–P). In this study, the findings suggested that ApoVs evoked calcium influx by fusing with CD8^+^ T cells, leading to the upregulated of L‐type voltage‐gated Ca^2+^ channels and subsequent calcium overload.

### 2.6 Harnessing Calcium Influx in CD8^+^ T Cells Weaken the Efficacy of ApoVs

**Figure 5:**
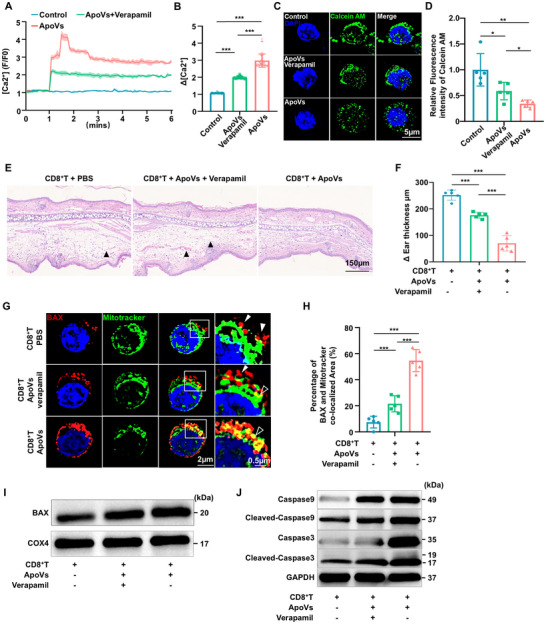
Harnessing Calcium Influx in CD8^+^ T Cells Weaken the Efficacy of ApoVs. A) Cytosolic Ca^2+^ levels and the mean maximal [Ca^2+^] rises (Δ[Ca^2+^]) in CD8^+^ T cells when stimulated with PBS, ApoVs, and ApoVs pretreatment with 1 µM verapamil groups in 6 min (*n* = 5). B) Quantification of the mean maximal [Ca^2+^] rises (Δ[Ca^2+^]) in CD8^+^ T cells after being treated with PBS, ApoVs, and ApoVs pretreatment with verapamil groups in 6 min (*n* = 60). C) SIM^2^ showed the Calcein AM expression in CD8^+^ T cells treated with PBS, ApoVs, and ApoVs pretreatment with verapamil respectively. Scale bar: 5 µm. D) Quantification of relative fluorescence intensity of Calcein AM in three groups (*n* = 5). E) H&E staining of ear samples collected 24 h after the challenge. Scale bar: 150 µm. Black arrowheads indicate dilated capillaries. F) Ear thickness was measured 24 h after the elicitation in groups of intravenous infusion of CD8^+^ T cells (*n* = 5). G) SIM^2^ showed the co‐localization of BAX (red) and Mitotracker (green) in three groups respectively. Scale bar: 2 µm. Boxed scale bar: 0.5 µm. H) Quantification of percentage of BAX and Mitotracker co‐localized Area (*n* = 5). I) Western blot displayed the expression level of BAX in isolated mitochondria from CD8^+^ T cells in three groups. J) Western blot displayed the expression level of classical apoptosis protein cleaved‐Caspase 9 and cleaved‐Caspase 3 in three groups. * *p *< 0.05, ** *p *< 0.01, ***p < 0.001.

To confirm the impact of calcium influx on CD8^+^ T cells, we employed verapamil, an inhibitor of L‐type Ca^2+^ channels. Fluo‐8‐AM calcium influx assay showed that ApoVs‐mediated intracellular calcium influx was reduced by verapamil treatment (**Figure** **5**A,B), suggesting that ApoVs‐induced intracellular calcium overload is partly dependent on the L‐type calcium channels. Calcein AM assay displayed increased fluorescence intensity after verapamil treatment, indicating that inhibiting calcium influx alleviated the mitochondrial membrane permeability (Figure 5C,D). Additionally, compared to the ApoVs group, the mitochondrial membrane potential increased and mitochondrial ROS levels decreased after verapamil treatment, implying that inhibiting calcium influx alleviated mitochondrial function (Figure S6A,B, Supporting Information). Histomorphology observations and measurements of relative ear thickness further confirmed that blocking calcium influx diminished the efficacy of ApoVs in treating CHS mice (Figure 5E,F).

To clarify whether ApoVs triggered the classical mitochondrial apoptotic cascades via L‐type Ca^2+^ channels, we conducted co‐localization staining and mitochondria isolation assay in the aforementioned experimental groups. The findings revealed a notable rise in the co‐localization of BAX and mitochondria (Mitotracker) in CD8^+^ T cells following ApoVs treatment. Furthermore, the co‐localization of BAX and Mitotracker was substantially diminished upon the inhibition of L‐type calcium channels using verapamil (Figure 5G,H). After mitochondria isolation, Western blot analysis confirmed an elevated level of BAX expression in mitochondria (Figure 5I). Meanwhile, Western blot analysis showed the inhibition of the calcium channel alleviated CD8^+^ T cells apoptosis with lower expression of specific apoptosis‐associated markers cleaved caspase‐9 and cleaved caspase‐3 (Figure 5J). Suggesting the translocation of BAX from cytoplasm to mitochondria, thereby initiating apoptosis in CD8^+^ T cells. These findings collectively suggest that ApoVs facilitate calcium overload through L‐type calcium channels, inducing mitochondria dysfunction, initiating apoptosis of CD8^+^ T cells, and ameliorating CHS responses.

### 2.7 ApoVs Treatment Ameliorated Oral Lichenoid Reaction

**Figure 6:**
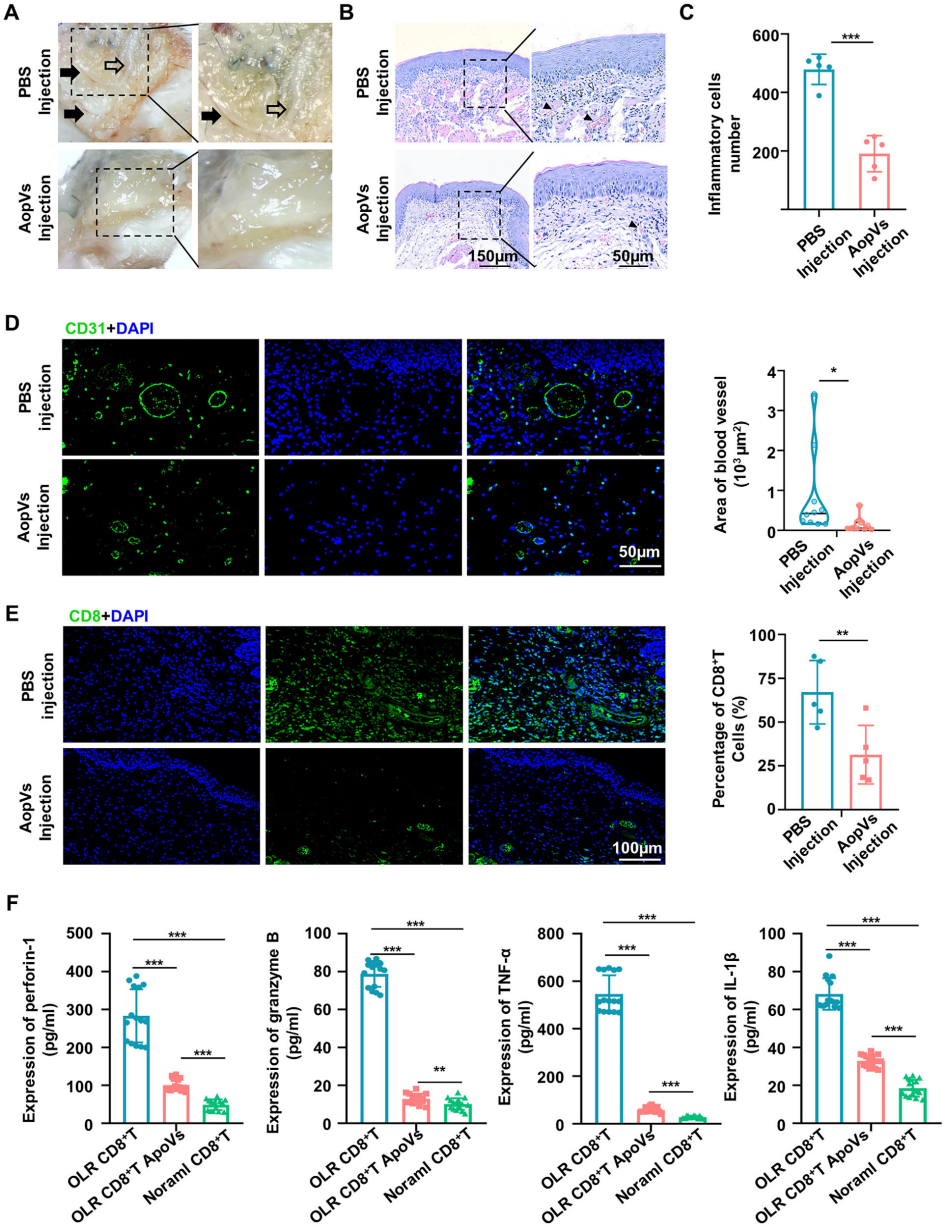
ApoVs Treatment Ameliorated Oral Lichenoid Reaction. A) Intraoral photo showed the lesion of oral lichenoid reaction with short white stripes in mice. The right panel magnifies the boxed area in the left panel. Black arrows indicate dilated blood vessels and white arrows indicate reticular white patches of oral mucosa. B) H&E staining of mice oral mucosa samples. The right panel magnifies the boxed area in the left panel. Scale bar: 150 µm for the left panel and 50 µm for the right panel. C) Quantification of H&E staining exhibited decreased inflammatory cells in lesion sites (*n* = 5). D) Immunostaining of mucosa lesions for CD31 (green) and DAPI (blue) showed the alleviated of dermal blood dilatation. Scale bar: 50 µm. Quantification of blood vessel area per section (*n* = 10). E) Representative images and quantification of CD8^+^ T cells in PBS and ApoVs injection groups (*n* = 5). Scale bar: 100 µm. F) The proinflammatory cytokines produced by CD8^+^ T cells in patients with OLR, as well as in the co‐cultured group of ApoVs and the control group, were assessed using ELSIA (*n* = 15). * *p *< 0.05, ** *p *< 0.01, ***p < 0.001.

To further validate the therapeutic efficacy of ApoVs in managing type IV hypersensitivity responses, we constructed an additional OLR murine model, which represents a type IV hypersensitivity response in pathology. During the elicitation phase, reticular stripes can be observed after the application of OXA to the buccal mucosa of mice (**Figure** **6**A). H&E staining revealed liquefaction degeneration of the basal keratinocytes and band‐like lymphocytic infiltration in the submucosal layer, closely resembling the features of oral lichen planus (OLP) (Figure 6B). However, in mice treated with ApoVs injection, these inflammatory manifestations were significantly alleviated (Figure 6A–C). Immunostaining of lesions displayed ApoVs injection alleviated the vascular remodeling and decreased expression of TNF‐α compared to PBS injection (Figure 6D, Figure S7A,B, Supporting Information). The percentage of CD8^+^ T cells also reduced after ApoVs injection, consistent with the histological findings (Figure 6E). More importantly, peripheral blood samples were collected from three healthy volunteers and three patients with OLR. CD8^+^ T cells were enriched and activated by CD3 and CD28 microbeads. Then, the CD8^+^ T cells from the patients with OLR were treated with ApoVs or PBS. The results indicated that in vitro treatment with ApoVs significantly reduced the levels of perforin‐1, granzyme B, TNF‐α, and IL‐1β secreted by the patient's CD8^+^ T cells (Figure 6F). Additionally, after being treated with ApoVs, the MPTP assay showed increased mitochondrial permeability in CD8^+^ T cells from patients (Figure S7C, Supporting Information), aligning with the CHS model. Therefore, our data suggested that ApoVs from MSCs can induce CD8^+^ T cell apoptosis and ameliorate CD8^+^ T cells mediated type IV hypersensitivity responses, shedding new light on preclinical and clinical research of MSCs‐derived ApoVs.

## 3 Discussion

In the present study, we dissect the novel function of SHED‐ApoVs in type IV hypersensitivity reactions for induction apoptosis of CD8^+^ T cells. MSCs‐ApoVs possess unique therapeutic functions in various diseases, including immune disorders, osteoporosis, skin injuries, and tumors. However, it remains elusive whether SHED‐ApoVs, acting as immune microenvironment modulators, possess immunomodulatory properties and interact with CD8^+^ T cells. This research utilized two models of type IV hypersensitivity reactions and identified for the first time the therapeutic role of SHED‐ApoVs in these reactions. Specifically, ApoVs promote membrane expression of L‐type voltage‐gated Ca^2+^ channels through membrane fusion in CD8^+^ T cells, leading to cytoplasmic calcium overload and fragmentation of mitochondria, subsequently induction of apoptosis in these cells. This study highlights the role of SHED‐ApoVs in inducing CD8^+^ T cell apoptosis and suggests their potential for treating CD8^+^ T cells‐mediated diseases, providing experimental evidence for nanovesicle‐based cell‐free therapies.

Apoptosis triggered by MSCs transplantation produces a specific quantity of ApoVs, a notable population of extracellular vesicles (EVs), which modulate the immune microenvironment.^[^ ^16^ ^]^ Based on their size, ApoVs can be categorized into three main subtypes: larger apoptotic bodies (1–5 µm), smaller apoptotic microvesicles (0.1–1 µm), and even more diminutive apoptotic exosomes (smaller than 150 nm).^[^ ^17^ ^]^ SHED as a type of MSCs possesses optimal immunomodulatory properties, engaging in immune remodeling and tissue regeneration processes.^[^ ^1^, ^18^ ^]^ It has been reported that SHED expresses PDL‐1 to ameliorate gland inflammation and dryness symptoms of Sjögren's syndrome by directly upregulating the ratio of Treg/Th17 via cell‐cell contact.^[^ ^9^ ^]^ Our team has previously demonstrated successful tooth regeneration in both animal models and clinical trials through the use of SHED, confirming their ideal anti‐inflammatory and regenerative properties.^[^ ^5^, ^8^, ^19^ ^]^^[^ ^2^ ^]^ However, there existed several challenges for stem cell therapy, which hindered the clinical practice, such as ethical considerations, tumor formation, immune rejection, substantial heterogeneity between donors, as well as batch‐to‐batch variation. ApoVs originating from MSCs serve as the modulator within the microenvironment and putatively mirror the biological function of their parent cells.^[^ ^2^ ^]^ For instance, SHED‐ApoVs enhanced the endothelial cells' angiogenic function and accelerated revascularization under the inflammatory microenvironment, promoting the efficient regeneration of dental pulp.^[^ ^10b^ ^]^ ApoVs derived from bone marrow mesenchymal stromal cells prevented Th17 differentiation and memory formation in the immune microenvironment, ameliorating joint erosion in murine arthritis.^[^ ^4^, ^20^ ^]^ MSCs‐ApoVs ameliorated the multiple myeloma in the tumor microenvironment by inducing multiple myeloma cell apoptosis.^[^ ^21^ ^]^ Due to the diverse and unique properties inherited from their parent cells, the exact biological characteristics of ApoVs from various sources and the mechanisms driving their physiological and therapeutic impacts remain incompletely elucidated. As for SHED‐ApoVs, their role as immune microenvironment modulators and their effects on immune cells are still largely unclear. Exploring the ApoV profile from diverse sources is beneficial for comprehending the significance of apoptosis in maintaining homeostasis and facilitates the advancement of cell‐free therapy employing nanovesicles. In this study, we first isolated and characterized SHED and SHED‐ApoVs as previously reported (Figure S1, Supporting Information). The findings confirmed the ApoVs obtained for this study are MSCs‐derived ApoVs.

CD8^+^ T cells mainly participate in combating viral infections and eliminating malignant cells in cancer.^[^ ^22^ ^]^ Nonetheless, when autoreactive responses occur and target the body's own cells, excessive activation of CD8^+^ T cells leads to autoimmune disorders and type IV hypersensitivity reactions.^[^ ^23^ ^]^ Type IV hypersensitivity is a class of diseases mediated by CD8^+^ T cells, including ACD^[^ ^13^ ^]^ and oral lichen planus.^[^ ^24^ ^]^ Type IV hypersensitivity also called delayed type hypersensitivity which primarily driven by CD8^+^ T cells, making it an ideal model for investigating the effects of ApoVs on these cells due to its distinct sensitization and elicitation phases. However, whether SHED‐ApoVs contribute to antihypersensitivity therapy has not been investigated. In this study, two type IV hypersensitivity disease models, CHS and OLR, were established and verified the therapeutic properties of SHED‐ApoVs on OXA‐induced tissue hypersensitivity responses (Figures 1A–D and 6A–D). ACD is a common skin disorder caused by external irritation that causes the skin to appear red, pimples, blisters, and pain.^[^ ^15^ ^]^ Alongside ACD, OLR represents type IV hypersensitivity responses associated with precancerous lesions, closely resembling the features of oral lichen planus.^[^ ^12b^ ^]^ Although several symptom‐based treatments are currently available to alleviate symptoms and manage the condition, there is a lack of established first‐line targeted treatment drugs. Developing effective immunotherapy and the realization of targeted therapy are the hot research issues for hypersensitivity responses. The function of CD8^+^ T cells was usually marked by the secretions of interferon γ (IFN‐γ), tumor necrosis factor α (TNF‐α), granzyme B, and perforin‐1.^[^ ^25^ ^]^ Additionally, it has been shown that CD8^+^ effector T cells produce IFN‐γ which boosts the production of IL‐1β and IL‐6 in allergen‐exposed sites and IFN‐γ also induces the production of chemokines like CXCL9, CXCL10 and CXCL11.^[^ ^11^ ^]^ This study showed that ApoVs treatment decreased the expression of pro‐inflammatory cytokines, suppressing tissue edema and hemangiectasis (Figure S2, Supporting Information). RNA sequencing of mice ear tissues implied the anti‐inflammatory and immunomodulatory function of SHED‐ApoVs, which is putatively related to apoptosis and metabolic process. Importantly, the local percentage of CD8^+^ T cells in tissue reduced after ApoVs treatment (Figures 1E–J and 6E). To further confirm the therapeutical effect of ApoVs, we collected a blood sample from a patient with oral lichen planus. The results suggested that ApoVs treatment significantly reduced the expression of CD8^+^ T cells function‐related cytokines, consisting with CHS findings (Figure 6F; Figures S3C–F and S7, Supporting Information). Thus, the results showed an anti‐inflammatory and immunomodulatory profile of ApoVs for alleviating CHS and OLR, implicating ApoVs serve as the immune microenvironment modulators in effectively attenuating the function and count of local CD8^+^ T cells.

Interest in EVs has progressively grown due to their potent role as microenvironment mediators locally and systemically.^[^ ^26^ ^]^ In addition, they can modulate the immune cells in a diversity paradigm including promoting macrophage M2 polarization,^[^ ^27^ ^]^ modulating Th17 cells,^[^ ^20^ ^]^ and generating neutrophil extracellular traps (NETs) formation.^[^ ^28^ ^]^ However, whether SHED‐ApoVs can modulate the function of CD8^+^ T cells remains to be elucidated. Our previous study utilized the adoptive transfer method to verify that the CHS were alleviated in WT mice which transferred with draining lymph node cells from OXA‐sensitized Sema4D KO mice.^[^ ^14^ ^]^ In the present study, we applied an adoptive transfer assay to transfer sensitized CD8^+^ T cells into unsensitized mice (Figure 2A–D). After 24 h of OXA treatment, there was no notable inflammation or ear thickening detected in wildtype recipient mice that did not undergo CD8^+^ T cell transfer. This observation indicated that the endogenous CD8^+^ T cells present in the wildtype recipient mice are incapable of mediating the local inflammation elicited by OXA. However, current research suggests that CD8^+^ T cells that are not specific for the antigen can be activated by cytokines without cognate antigens, a phenomenon termed bystander T cell activation.^[^ ^29^ ^]^ It is possible that during the OXA‐induced inflammation, bystander CD8^+^ T cells from wildtype recipient mice might also participate in the process, which will be explored in future studies. Collectively, the results uncovered that ApoVs treatment directly attenuated CHS response via promoting apoptosis of CD8^+^ T cells (Figure 2).

ApoVs can promote the apoptosis of several types of immune cells and maintain or remodel immune homeostasis through various mechanisms.^[^ ^30^ ^]^ Exploring the mechanism of ApoVs on the immune system is helpful to understand the significance of apoptosis in tissues and organs under the homeostasis condition, and is conducive to the construction of cell‐free therapy based on nanovesicles. Recently, ApoVs, microvesicles (MVs), small EVs, and exosomes have been identified as carriers of both damaged and intact mitochondria, as well as mitochondrial components, facilitating intercellular mitochondrial transfer.^[^ ^31^ ^]^ This transfer can lead to significant effects on the recipient cells, such as mitophagy, metabolic reprogramming, and changes in mitochondrial dynamics.^[^ ^32^ ^]^ The study has shown that co‐culturing bone marrow stromal cells (BMSCs) with CD8^+^ T cells enhances the antitumor efficacy of the latter through tunneling nanotubes‐mediated mitochondrial transfer from BMSCs. However, when CD8^+^ T cells were co‐cultured with free‐floating mitochondria derived from BMSCs, no significant mitochondrial uptake was observed.^[^ ^33^ ^]^ In this study, proteomic sequencing results indicate that SHED‐ApoVs contain mitochondrial components (Figure 3A,B), yet it is unclear whether these vesicles harbor intact, healthy, and functional mitochondria. Given that ApoVs encompass a wide variety of substances, such as nucleic acids, proteins, and lipids, it is essential to employ MicroRNA sequencing, lipidomics, and metabolomics to thoroughly analyze their compositions and elucidate the specific mechanisms. Previous proteomic sequencing of MSC‐derived ApoVs identified apoptotic signatures from parent cells, including Fas, SOD1, and Caspase family members, which induce apoptosis in recipient cells.^[^ ^34^ ^]^ During apoptosis, the loss of mitochondrial membrane potential and caspase expression can occur in the early stage, while the loss of membrane integrity occurs in the late stage.^[^ ^35^ ^]^ Thus, the depolarization of mitochondrial membrane potential is a landmark event during the initial phase of apoptosis. Fragmentation of mitochondria is a multifaceted manifestation of various forms of mitochondrial dysfunction. It reflects disturbances in mitochondrial dynamics, membrane potential, energy metabolism, oxidative stress, and calcium homeostasis.^[^ ^36^ ^]^ Notably, mitochondrial dysfunction in apoptotic cells typically manifests through alterations in morphology, ROS accumulation, and permeability enhancement.^[^ ^37^ ^]^ In this study, we showed that the fragmentation of mitochondria with elevated mitochondrial permeability, declined mitochondrial membrane potential and ROS accumulation, implying the dysfunction of mitochondria (Figure 3C–J). The findings above suggest that ApoVs induced apoptosis of CD8^+^ T cells by impairing the function of mitochondria.

EVs can transmit information by acting at the cell surface, without content delivery. For example, it has been reported that apoEVs directly contact multiple myeloma cells to facilitate Fas trafficking from the cytoplasm to the cell membrane, leading to the initiation of apoptosis.^[^ ^21^ ^]^ Given the limited phagocytic capacity of T cells, their interaction patterns and mechanisms with EVs differ from those observed with macrophages and MSCs, which typically internalize EVs through endocytosis or phagocytosis.^[^ ^38^ ^]^ It has been reported that EVs derived from dendritic cells can bind to clusters of CD8 molecules on the surface of CD8^+^ T cells, thereby boosting the responses of effector CD8^+^ T cells during viral infections.^[^ ^39^ ^]^ However, studies clarifying how MSCs derived‐ApoVs influence T cells, especially CD8^+^ T cells, are still lacking. In this study, we unexpectedly found that ApoVs interact with CD8^+^ T cells in a membrane fusion manner (Figure 4A–C; Figure S5, Supporting Information). To our knowledge, this kind of membrane‐based communication manner has rarely been reported in ApoVs influence on T cells. Proper cellular functions rely on the precise regulation of intracellular Ca^2+^ levels.^[^ ^40^ ^]^ It has been reported that ApoVs directly contact the membrane of multiple myeloma cells, evoking Ca^2+^ influx and elevating cytosolic calcium levels.^[^ ^21^ ^]^ Therefore, we analyzed the levels of calcium after ApoVs interacted with CD8^+^ T cells in the short and long term, suggesting that ApoVs induced persistent calcium overload of CD8^+^ T cells with cell membrane fusion (Figure 4D–G), shedding new lights on membrane‐based communication between ApoVs and T cells. In future studies, immunogold staining, recognized as the gold standard for demonstrating the presence of SHED‐ApoVs—distinct from EVs secreted by CD8^+^ T cells—on the surface of CD8^+^ T cells, should be employed to enhance the credibility of findings.

L‐type voltage‐gated Ca^2+^ channels, such as Ca_v_1.1, Ca_v_1.2, and Ca_v_β1, have been shown to modulate Ca^2+^ signaling and contribute to T cell function.^[^ ^41^, ^42^ ^]^ Previous research has highlighted the critical role of Ca_v_1.2 channels in the Ca^2+^ response and cytokine production of human Th2 lymphocytes stimulated by the T cell receptor.^[^ ^43^ ^]^ Additionally, Ca_v_β1 has been proved as a critical regulator in T cell activities including expansion and apoptosis.^[^ ^44^ ^]^ Calcium homeostasis is crucial for maintaining, and disruptions in calcium balance, particularly calcium overload, can induce morphological changes such as mitochondrial swelling and fragmentation.^[^ ^40^, ^45^ ^]^ In this study, we showed that the increased expression of L‐type Ca^2+^ channels proteins on the membrane of CD8^+^ T cells with calcium overloaded, resulting to the initiation of apoptosis (Figure 4H–P). More importantly, verapamil treatment, which inhibits L‐type voltage‐gated Ca^2+^ channels, has been shown to partially restore mitochondrial permeability, harness calcium influx, and diminish the efficacy of ApoVs on the CHS response (Figure 5A–F; Figure S6, Supporting Information). Studies have indicated that mitochondrial dysfunction initiates classical apoptotic biochemical cascades by mitochondrial permeability transition and translocation of pro‐apoptotic BAX to mitochondria. This leads to the release of cytochrome C from mitochondria to the cytosol, activating the apoptogenic molecules such as cleaved‐caspase 9 and cleaved‐caspase 3.^[^ ^46^ ^]^ Therefore, we examined and confirmed the translocation of BAX from cytoplasm to mitochondria, initiating apoptosis CD8^+^ T cells which were rescued by inhibitor of L‐type voltage‐gated Ca^2+^ channels (Figure 5G–J). These findings implicated that membrane fusion elevated the membrane permeability of CD8^+^ T cells via L‐type voltage‐gated Ca^2+^ channels, providing a novel role of L‐type voltage‐gated Ca^2+^ channels in CD8^+^ T cells. Additionally, the targeted elimination of endogenously generated ApoVs may offer therapeutic potential for treating diseases associated with reduced calcium influx, such as cardiovascular diseases, neurodegenerative diseases, diabetes, and others. However, whether ApoVs have similar functions in other cell types requires further exploration.

Autoreactive CD8^+^ T cells have been implicated in the pathogenesis of multiple autoimmune diseases, including Crohn's disease, rheumatoid arthritis, and multiple sclerosis. Pathogenic CD8^+^ T cells express high levels of cytotoxic effector molecules such as IFN‐γ, TNF, granzyme B, and perforin.^[^ ^47^ ^]^ However, during chronic viral infections or cancer, CD8^+^ T cells are in a dysfunctional state which is referred to as CD8^+^ T cell exhaustion. It is characterized by a gradual loss of effector functions and an inability to effectively respond to antigens, leading to compromised immune surveillance and host defense.^[^ ^48^ ^]^ In this study, we demonstrated ApoVs treatment inducing apoptosis of CD8^+^ T cells, suggesting that timely clearance of ApoVs could be beneficial for CD8^+^ T cells in anti‐viral and anti‐tumor therapy. Furthermore, the significant role of L‐type voltage‐gated Ca^2+^ channels during the interaction of ApoVs with CD8^+^ T cells indicates their potential as drug targets for exhausted CD8^+^ T cells in antiviral and antitumor therapy.

## 4 Conclusion

Apoptosis is a vital process in maintaining the homeostasis of multicellular organisms. Despite limited understanding of the precise functions of ApoVs in various pathological conditions and cell types, our research has revealed that MSCs‐ApoVs can induce apoptosis in CD8^+^ T cells, thereby ameliorating CD8^+^ T cell‐driven type IV hypersensitivity reactions such as CHS and OLR. Notably, analysis of blood samples from a patient with OLR confirmed the anti‐hypersensitivity properties of ApoVs by modulating CD8^+^ T cell activity. Upon interaction with CD8^+^ T cells, ApoVs were observed to merge their membrane with that of the CD8^+^ T cells, triggering calcium influx and mitochondrial dysfunction, ultimately leading to CD8^+^ T cell apoptosis. These findings offered a new insight into the interaction of MSCs‐ApoVs and CD8^+^ T cells, demonstrating that MSC‐ApoVs, as nano‐mediators in the immune microenvironment, have the potential to treat CD8^+^ T cell‐mediated hypersensitivity. Our findings indicated that products from apoptotic cells putatively impede anti‐tumor or anti‐viral treatment, providing a promising avenue for developing therapeutic interventions targeting CD8^+^ T cells participated diseases.

## 5 Experimental Section

### Oxazolone‐induced Contact Hypersensitivity and Oral Lichenoid Reactions

Mice models were established following the guidelines of the Ethical Committee of the School of Stomatology, Fourth Military Medical University (license number: kq‐2024‐016). were sensitized through topical application of 100 µL of 3.0% oxazolone (OXA, dissolved in anhydrous ethanol, MedChemExpress, USA) on the shaved abdomen. After a period of five days, mice were subjected to contact hypersensitivity by being challenged with 10 µL of 1% OXA or ethanol on both sides of one ear. For oral lichenoid reactions, 50 µL of 1% OXA or ethanol was applied to the buccal mucosa. The ear thickness before and after elicitation was measured using an engineer's micrometer caliper. The difference in the increase in thickness between the left and right ears was analyzed. The macroscopic and dermatoscopic appearances of the lesions were recorded using a dermatoscope (Illuco IDS 1100; South Korea).

### Histology Assay

Briefly, ear or mucosal tissues were collected, fixed in 4% paraformaldehyde for 12 h, dehydrated with graded ethanol, and embedded in paraffin. The samples were sectioned at a thickness of 4 µm thickness and stained with hematoxylin and eosin (H&E) (Leica, Germany). The images were captured using a fluorescence continuous scanning digital section microscope with a full slide scanning system (VS200, Olympus, Japan).

### Immunohistochemistry and Immunofluorescence Staining

Immunohistochemistry (IHC) staining was performed on 4 µm paraffin‐embedded sections of the ear and mucosal tissues. The sections underwent dewaxing and hydration steps, followed by heat‐mediated antigen retrieval using Tris–EDTA (C1038, Solarbio, China) in a pressure cooker. Time the pressure cooker once it reaches pressure and seal it after 100s. After gently washing the sections three times in PBS, 3% hydrogen peroxide was administered at 37 °C for 1 h. To block nonspecific antigens, 5% bovine serum albumin (BSA, FC0077, MP Biomedicals, USA) was used. Primary antibodies were incubated overnight at 4 °C, while fluorescent secondary antibodies were incubated for 1 h at room temperature. Sections were sealed with a mounting medium containing 4,’‐6‐diamidino‐2‐phenylindole (DAPI; ab104139, Abcam, UK) after being washed with PBS 3 times, and images were captured by fluorescence continuous scanning digital section microscope with full slide scanning system (VS200, Olympus, Japan) and analyzed by ImageJ software.

Immunocytochemistry (ICC) staining was performed on CD8^+^ T cells. CD8^+^ T cells were prelabeled with PKH67 using PKH67 Green Fluorescent cell Linker Kit (MIDI67, Sigma‐Aldrich, USA) and cultured with PKH26 (MIDI26, Sigma‐Aldrich, USA) prelabeled ApoVs (20 µg mL^−1^) for 6 h. After Fluo‐8 AM or Mito‐track staining, CD8^+^ T cells were fixed with 2.5% glutaraldehyde (111‐30‐8, Macklin, China). Then, the primary antibodies were incubated overnight at 4 °C, while fluorescent secondary antibodies were incubated for 1 h at room temperature, and the nucleus was stained with Hoechst 33 342 (ab228551, Abcam, UK, diluted 1:50). Images were captured by Lattice doubling the resolution of structured illumination microscopy (SIM^2^) (Elyra 7, ZEISS, Germany) and analyzed by ImageJ software.

The antibodies used were: anti‐CD31 antibody (FAB3628G, R&D Systems, USA; diluted 1:100), anti‐TGF‐β antibody (3711S, Cell Signaling Technology, USA, diluted 1:200), anti‐TNF‐α antibody (11948s, Cell Signaling Technology, USA, diluted 1:200), mouse CD8 alpha Alexa Fluor 488 MAb (FAB116G‐100, R&D system, USA, diluted 1:200), anti‐Ca_v_1.2 antibody (ACC‐003, Alomone, Israel, diluted 1:200), anti‐Ca_v_β1 antibody (PA5‐109285, Invitrogen, USA, diluted 1:200), anti‐BAX antibody(60267‐1‐Ig, Proteitech, China, diluted 1:200), Alexa Fluor 488 Donkey anti‐Mouse secondary antibody (A21202, Invitrogen, USA, diluted 1:200), Alexa Fluor 594 Donkey anti‐Mouse secondary antibody (A21203, Invitrogen, USA, diluted 1:200), Alexa Fluor 488 Donkey anti‐Rabbit secondary antibody (A21206, Invitrogen, USA, diluted 1:200) and Alexa Fluor 594 Donkey anti‐Rabbit secondary antibody (A21207, Invitrogen, USA, diluted 1:200).

### PKH26 and PKH67 Staining

To detect the behavior of ApoVs or CD8^+^ T cells in vitro and in vivo, ApoVs or CD8^+^ T cells was labelled with the PKH26 Red Fluorescent Cell Linker Kit (MIDI26, Sigma‐Aldrich, USA) and the PKH67 Green Fluorescent Cell Linker Kits (MIDI67, Sigma‐Aldrich, USA) following the manufacturer`s instructions. 200 ug ApoVs or 2 × 10^5^ CD8^+^ T cells were collected and washed once using medium without serum. Subject the ApoVs to centrifugation at 16 000 × g for a duration of 30 min, and the CD8^+^ T cells to centrifugation at 400 × g for 5 min to form a loose pellet. Subsequently, carefully aspirate the supernatant, ensuring that no more than 25 µL remains. Adding 1 mL of Diluent C to the ApoVs pellet and resuspending with gentle pipetting to ensure complete dispersion. Then, 4 uL of fluorescent dye PKH26/PKH67 ethanolic dye solution was added and incubated at room temperature for 5 min. A volume of 2 mL of fetal bovine serum was added, followed by centrifugation at 16 000 × g for 30 min to isolate ApoVs, and at 400 × g for 10 min for CD8^+^ T cells, both conducted at 4 °C to remove the free PKH26/PKH67 dye. The resulting precipitates were subsequently resuspended in PBS for further analysis.

### Adoptive Transfer Assessment

Adoptive transfer of lymph node cells was performed as previously described with modifications.^[^ ^12^ ^]^ In brief, 6 to 8‐week‐old male wildtype mice were sensitized with OXA. Five days after sensitization, inguinal lymph node cells were isolated to prepare single‐cell suspensions. CD8^+^ T cells were extracted and treated with ApoVs (10 µg mL^−1^), PBS, or Verapamil (1 µM, 52‐53‐9, MedChemExpress, USA) for 6 h. Then, unsensitized mice were injected intravenously with 5 × 10^7^ CD8^+^ T cells. Two hours later, baseline ear thickness was measured, and the mice were then challenged with 10 µL of 1.0% OXA on both sides of one ear. Ear thickness was measured 24 h later, and the increase in ear thickness above baseline was calculated.

### CD8+ T Cells Isolation and Culture

C57 mice (6‐8 weeks old, male, Air Force Military Medical University, Xi'an) were anesthetized with 1% pentobarbital sodium (50 mg kg^−1^ body weight) and sacrificed by spinal cord dislocation. Spleens were removed and ground using a sterile plunger, and then the spleen cells were isolated through a 70 µm cell strainer. Centrifuge at 800 rpm for 5 min and discard the supernatant. Resuspend and lyse cells in red blood cell lysis buffer for 20 min at 4 °C. The samples were centrifuged at 800 rpm for 5 min and resuspended in a solution prepared by diluting Magnetic‐Activated Cell Sorting (MACS) BSA Stock Solution at a ratio of 1:20 with autoMACS Rinsing Solution. CD8^+^T lymphocytes were magnetically labeled with CD8 (TIL) MicroBeads. Then cell suspension was then loaded onto a MACS Column, which was placed in the magnetic field of a MACS Separator. After removing the column from the magnetic field, the magnetically retained CD8^+^ cells were eluted as the positively selected cell fraction. Total positively isolated CD8 ^+^ T cells were stimulated using plate‐bound anti‐CD3 (2 µg mL^−1^) plus soluble anti‐CD28 (1 µg mL^−1^) monoclonal antibodies for two days and cultured in RPMI 1640 medium (Gibco, USA) supplemented with 10% fetal bovine serum (FBS, Gibco, USA).

Human CD8^+^ T cells were isolated following the guidelines of the Ethical Committee of the School of Stomatology, Fourth Military Medical University (license number: KQ‐YJ‐2023‐164). Three healthy volunteers and three patients with oral lichen planus were informed of the objectives of this research, and written informed consent was obtained prior to conducting the study. In detail, mononuclear cells were obtained from the peripheral blood of healthy individuals and patients with oral lichen planus using Ficoll‐Paque PREMIUM (17 544 202, GE Life). Human CD8^+^ T cells were magnetically labeled with CD8 MicroBeads (130‐045‐201, Miltenyi Biotec) and isolated as mentioned above.

### Apoptosis Assay

The Annexin V‐FITC Apoptosis Detection Kit (BMS500FI‐100, ThermoFisher Scientific, USA) was utilized following the manufacturer's protocol. Annexin‐V fluorescein isothiocyanate (FITC) and propidium iodide (PI) combined working solution was prepared to examine the apoptotic CD8^+^ T cells. Cells collected were incubated with the working solution for 15 min at 4 °C in the dark. After staining, cells were analyzed using a flow cytometer, and the number of Annexin V‐positive cells was determined.

### RNA Extraction and Sequencing

The total RNA from the two groups of mice ears after 24 h of ApoVs treatment (20 mg mL^−1^) was extracted following the instruction manual of the TRIzol Reagent (Life Technologies, California, USA). After measuring the RNA concentration and purity, 1 µg of RNA per sample was used as input material for the RNA sample preparations. Sequencing libraries were generated using the Hieff NGS Ultima Dual‐mode mRNA Library Prep Kit for Illumina (Yeasen Biotechnology) following the manufacturer's recommendations, and index codes were added to attribute sequences to each sample. The libraries were sequenced on an Illumina NovaSeq platform to generate 150 bp paired‐end reads, following the manufacturer's instructions. Differentially expressed genes (DEGs) were identified in the ApoVs injection group compared to the control group. These genes were defined as those with a fold change ≥1.5 and a false discovery rate (FDR) < 0.05. They were included for further analysis based on the Gene Ontology (GO) database. GO of biology process enrichment analysis of the up‐ and down‐regulated genes was implemented using the clusterProfiler package in RStudio software (version 4.3.1). Among the top 10 gene enrichment pathways, those with a *p* value < 0.05 were exhibited.

The total RNA from the CD8^+^ T cells of mice (*n* = 3) was extracted after 6 h ApoVs treatment (10 ug mL^−1^) and sequenced as mentioned above. DEGs were identified and defined as those with a fold change ≥2 and a FDR < 0.05. A total of 322 upregulated genes were included for further analysis based on the KEGG and Reactome databases. ClusterProfiler package was used to analyze the upregulated genes in KEGG pathways using RStudio software (version 4.3.1). Reactome pathway enrichment analysis was performed using the Reactome pathway database (https://reactome.org/). Top 10 KEGG gene enrichment pathways with FDR < 0.05 were visualized in bubble plots, and the top 12 Reactome gene enrichment pathways with FDR < 0.05 were also displayed.

### Proteomic Analysis

Proteins from SHED‐derived ApoVs and SHED‐derived EVs were extracted to obtain peptides, which were then analyzed using LC‐MS/MS analysis on a NanoElute UHPLC system (Bruker Daltonics, USA). The raw data was analyzed using the MaxQuant search engine (v.1.6.15.0). Differentially expressed proteins (DEPs) were defined as those with a fold change ≥2 and FDR < 0.05 and were included for further analysis based on GO and Reactome Pathway databases. The top 10 enrichment pathways with an FDR < 0.05 were displayed in bubble and bar plots.

### Western Blot Analysis

Protein samples of ApoVs and CD8^+^ T cells were analyzed as previously reported.^[^ ^49^ ^]^ The antibodies used were: anti‐CD9 antibody (#A19027, Abclonal, China, diluted 1:1000), anti‐CD63 antibody (#sc‐5275, Santa Cruz Biotechnology, USA, diluted 1:1000), anti‐TSG101 antibody (#ET1701‐59, Huabio, China, diluted 1:1000), anti‐GM130 antibody (#610 822, BD Pharmingen, USA, diluted 1:1000), anti‐Caspase9 antibody (#9504, Cell Signaling Technology, USA, diluted 1:1000), anti‐Cleaved Caspase9 antibody (#9509, Cell Signaling Technology, USA, diluted 1:1000), anti‐Caspase3 antibody (#9662, Cell Signaling Technology, USA, diluted 1:1000), anti‐Cleaved Caspase3 antibody (#9664, Cell Signaling Technology, USA, diluted 1:1000), anti‐Ca_v_1.1 antibody (ACC‐314, Alomone, Israel, diluted 1:1000), anti‐Ca_v_1.2 antibody (ACC‐003, Alomone, Israel, diluted at 1:1000), anti‐Cavβ1 antibody (PA5‐109285, Invitrogen, USA, diluted 1:1000), anti‐BAX antibody (60267‐1‐Ig, Proteitech, China, diluted 1:1000), anti‐GAPDH antibody (YM3029, Immunoway, USA, diluted 1:1000) and anti‐COX4 antibody (#4850S, Cell Signaling Technology, USA, diluted 1:1000). Goat anti‐Mouse secondary antibody (115‐035‐003, Jacson, USA, diluted 1:1000), Goat anti‐Rabbit secondary antibody (111‐035‐003, Jacson, USA, diluted 1:1000) and Rabbit anti‐Goat secondary antibody (305‐035‐003, Jacson, USA, diluted 1:1000).

### Scanning Electron Microscopy (SEM)

CD8^+^ T cells were co‐cultured with SHED‐ApoVs (method as before) for 1, 2, 3, 4, and 6 h separately. Then the cells were added onto the surface of coverslips which were coated with Poly‐D‐Lysine (R30150,Yuan Ye, China) for 1 h. Then the cells were fixed with 2.5% glutaraldehyde for 30 min, dehydrated with graded ethanol solutions (50%, 70%, 95%, 100%, 5 min each), and dried with hexamethyldisilane. The dried samples were coated with gold using a sputter coater and observed under SEM to evaluate the fusion of SHED‐ApoVs with the cell membranes of CD8^+^ T cells.

### Transmission Electron Microscopy (TEM)

CD8^+^ T cells from the spleen of mice were fixed with 3% glutaraldehyde at 4 °C for 5 min. Afterward, CD8^+^ T cells were centrifuged at 12 000 rpm for 10 min to remove the supernatant. Resuspend the deposit with 3% glutaraldehyde at 4 °C for further examination. The samples were applied to copper grids (BZ11022a, EMCN, China) and stained with 2% phosphotungstic acid (GZ02536, Ruixin, China) using standard methods. Stained grids were examined using a transmission electron microscope (JEM‐1400FLASH, JEOL, Japan). Mitochondrial morphology in CD8^+^ T cells was observed, and the abnormal mitochondria were defined as those with a loose cristae structure and a brighter appearance.

### Mitochondrial Morphological Measurements

The groups of CD8^+^ T cells, with or without ApoVs treatment (10 ug mL^−1^), were plated at 0.5 × 10^6^ cells mL^−1^ in 1.5 mL centrifuge tubes. Mitochondrial morphological measurements were conducted using MitoTracker Green FM (M7514, Invitrogen, USA) following the manufacturer's instructions and were observed using the Lattice doubling the resolution of structured illumination microscopy (SIM^2^) (Elyra 7, ZEISS, Germany). The perimeter and area of individual mitochondrial branches were traced using ImageJ software, and the mean perimeter and area of mitochondria per cell were quantified.

### Mitochondrial Permeability Measurement

Cells from different groups were plated at 10^6^ cells mL^−1^ in 1.5 mL centrifuge tubes. Mitochondrial permeability was assessed using the MPTP Assay Kit (C2009S, Beyotime, China) following the manufacturer's instructions. Then cells were then plated at 10^4^ cells mL^−1^ in a confocal dish (1 mL dish^−1^) that was treated with Poly‐D‐Lysine (Gibco, USA). After a 30‐minute treatment, cells were incubated with Hoechst 33342 (ab228551, Abcam, UK, diluted 1:50), and images were captured using Lattice SIM^2^ (Elyra 7, ZEISS, Germany). The relative fluorescence intensity of Calcein AM was analyzed using the ImageJ software. Furthermore, the stained cells were analyzed using flow cytometry (BD Biosciences, USA), and the fluorescence intensity of Calcein AM was assessed with FlowJo software.

### Intracellular Calcium Measurements

Cytosolic Ca^2+^ was measured using Fluo‐8AM (ab142773, abcam, UK). For instantaneous Ca^2+^ measurement: After pretreatment with verapamil (1 µM, 52‐53‐9, MedChemExpress, USA) or PBS for 30 min, CD8^+^ T cells were incubated with 5 µM Fluo‐8‐containing buffer solution for 30 min at 37 °C, then washed by Hanks Balanced Salt Solution with 20 mM HEPES (HHBBS) (2611S231799, maokangbio, China). Next, 5 × 10^4^ cells per each well were seeded to 96‐well black plates in HHBS containing ApoVs (10 µg mL^−1^) and 2 mM CaCl_2_ (ST365, Beyotime, China). BioTek fluorescence microplate reader (Bioteck, USA) was applied to detect intracellular Ca^2+^ for 6 min by exciting at 490 nm and detecting emission at 514 nm. For long‐term Ca^2+^ measurement: The cells were cocultured with ApoVs (10µg mL^−1^) and 2 mM CaCl_2_ for 6 h and incubated with 5 µM Flou‐8‐containing buffer solution for 30 min at 37 °C and washed by HHBBS. Then, 5 × 10^4^ cells per well were seeded to 96‐well black plates. Intracellular calcium fluorescence was detected by BioTek fluorescence microplate reader as above mentioned. Cytosolic Ca^2+^ changes were presented as fluorescence intensity relative to baseline fluorescence over time: F/F0, in which F represents the fluorescence intensity measured and F0 represents the minimum fluorescence intensity.

### Mitochondria Isolation

Mitochondria isolation was performed using the Cell Mitochondria Isolation Kit (89 874, Thermo Scientific, USA) according to the manufacturer's instructions. The protein of isolated mitochondria was collected, COX4 were applied as mitochondrial reference protein and BAX expression level was analyzed by Western blot.

### Membrane and Cytosol Protein Extraction

Membrane and cytosol protein of CD8^+^ T cells were extracted using Membrane and Cytosol Protein Extraction Kit (20127ES50, Yeasen, China) following the manufacturer's instructions. The expression levels of Ca_v_1.1, Ca_v_1.2, and Ca_v_β1 of membrane and cytosol were analyzed by Western blot respectively.

### Enzyme‐Linked Immunosorbent Assay (ELISA)

The following reagents were used according to the manufacturer's instructions for detection in serum and culture supernatants: Mouse IFN‐γ ELISA Kit (EMC101g.96, Neobioscience, China), Mouse TNF‐α ELISA Kit (EMC102a.96, Neobioscience, China), Mouse Perforin‐1 ELISA Kit (SEB317Mu, Cloud Clone, China), Mouse Granzyme B ELISA Kit (SEA600Mu, Cloud Clone, China), Human TNF‐α ELISA Kit (EHC103a.48, Neobioscience, China), Human IL‐1β ELISA Kit (EHC002b.96, Neobioscience, China), Human Perforin‐1 ELISA Kit (SEB317Hu, Cloud Clone, China), Human Granzyme B ELISA Kit (SEA600Hu, Cloud Clone, China).

### JC‐1 Assay

Cells were plated at 1 × 10^6^ cells mL^−1^ in black 96‐well plates (100 µL per well). Mitochondrial membrane potential was measured with JC‐1 kit (C2006, Beyotime, China), following the provided guidelines. The stained cells were analyzed by BioTek fluorescence microplate reader (Bioteck, USA).

### Mitochondria ROS Measurement

Cells were plated at 1 × 10^6^ cells mL^−1^ in black 96‐well plates (100 µL per well). Mitochondria ROS levels were measured with MitoSOX Red kit (HY‐D1055, MCE, USA), following the provided guidelines. The stained cells were analyzed by BioTek fluorescence microplate reader (Bioteck, USA).

### Statistical Analysis

GraphPad Prism (version 8.0) was utilized for statistical analysis. The two‐tailed Student's t‐test was applied to assess the differences in means between the two groups.

## Conflict of Interest

The authors declare no conflict of interest.

## Supporting information



Supporting Information

## Data Availability

The data that support the findings of this study are available from the corresponding author upon reasonable request.
