# Supplementary material for: Apoptotic Vesicles Derived from Mesenchymal Stem Cells Ameliorate Hypersensitivity Responses via Inducing CD8+ T Cells Apoptosis with Calcium Overload and Mitochondrial Dysfunction
Source: Adv Sci (Weinh). 2025 Mar 16;12(22):2407446. doi: 10.1002/advs.202407446 (PMC12165088; doi:10.1002/advs.202407446)
Supplement: Supplementary file 1 — Supporting Information [file ADVS-12-2407446-s001.docx]

Supporting Information

**Apoptotic Vesicles Derived from Mesenchymal Stem Cells Ameliorate Hypersensitivity Responses via inducing CD8^+^ T Cells Apoptosis with Calcium Overload and Mitochondrial Dysfunction**

*Anqi Liu^1,2#^, Peng Peng^1#^, Changze Wei^4#^, Fanhui Meng^1^, Xiaoyao Huang^1^, Peisheng Liu^1^, Siyuan Fan^1^, Xinyue Cai^1^, Meiling Wu^1^, Zilin Xuan^5^, Qing Liu^3^, Xinyu Qiu^1*^, Zhenlai Zhu**^3*^, Hao Guo^1*^*

^1^ State Key Laboratory of Oral & Maxillofacial Reconstruction and Regeneration, National Clinical Research Center for Oral Diseases, Shaanxi Clinical Research Center for Oral Disease, Department of Preventive Dentistry, School of Stomatology, The Fourth Military Medical University, Xi’an, Shaanxi 710032, China.

^2^ Department of Stomatology, 985 Hospital of Joint Logistics Support Force, Taiyuan, Shanxi 030000, China.

^3^ State Key Laboratory of Oral & Maxillofacial Reconstruction and Regeneration, National Clinical Research Center for Oral Diseases, Shaanxi Clinical Research Center for Oral Disease, Department of Oral Medicine, School of Stomatology, The Fourth Military Medical University, Xi'an, Shaanxi 710032, China.

^4^ Department of Chemical and Biomolecular Engineering, National University of Singapore, 4 Engineering Drive 4, Singapore 117585.

^5^ Faculty Of Medicine And Health, University of Sydney, Camperdown NSW 2050, Australia.

^#^ These authors contributed equally to this study.

*** Corresponding authors**

**Hao Guo** − State Key Laboratory of Oral & Maxillofacial Reconstruction and Regeneration, National Clinical Research Center for Oral Diseases, Shaanxi Clinical Research Center for Oral Disease, Department of Preventive Dentistry, School of Stomatology, The Fourth Military Medical University, 145 West Changle Road, Xi’an, Shaanxi 710032, China. orcid.org/ 0000-0003-3720-4480; E-mail: [guohaofmmu@163.com](mailto:guohaofmmu@163.com).

**Zhenlai Zhu** − State Key Laboratory of Oral & Maxillofacial Reconstruction and Regeneration, National Clinical Research Center for Oral Diseases, Shaanxi Clinical Research Center for Oral Disease, Department of Oral Medicine, School of Stomatology, The Fourth Military Medical University, Xi'an, Shaanxi 710032, China.

E-mail:zhenlai_zhu@163.com.

**Xinyu Qiu** − State Key Laboratory of Oral & Maxillofacial Reconstruction and Regeneration, National Clinical Research Center for Oral Diseases, Shaanxi Clinical Research Center for Oral Disease, Department of Preventive Dentistry, School of Stomatology, The Fourth Military Medical University, 145 West Changle Road, Xi’an, Shaanxi 710032, China. E-mail: qiuxinyu@fmmu.edu.cn.

**KEYWORDS：**apoptotic vesicles, type IV hypersensitivity responses, CD8^+^ T cells, extracellular vesicle, mesenchymal stem/stromal cells

**MATERIALS AND METHODS**

**Isolation and Characterization of SHED and SHED-ApoVs.**

SHED were isolated and identified as previously reported. ^[1]^ Briefly, dental pulp from human exfoliated deciduous teeth was collected from the School of Stomatology of the Fourth Military Medical University with the approval of the Ethical Committee of the School of Stomatology, the Fourth Military Medical University (license number: KQ-YJ-2023-164). SHED were cultured in alpha modification of Eagle's medium (α-MEM, Gibco, United States) with 10% fetal bovine serum (FBS, Gibco, USA). Colony-forming unit assay was used to demonstrate the self-renewal potential, while osteogenic and adipogenic induction were assessed using alizarin red and oil red O staining to show the multiple differentiation capabilities of SHED.

SHED-ApoVs were isolated and identified as previously reported.^[2]^ Specifically, SHED were treated with staurosporine (HY-15141, MedChemExpress, 100 nM) for 16 hours to induce apoptosis. Then, the culture media were collected and centrifuged at 800 g for 10 min to remove the cells, and at 2000 g for 10 min to remove the debris. Then, the supernatant was further centrifuged at 16000 g for 30 min at 4 ℃ to concentrate the ApoVs in the pellet. Next, the pellet was suspended in PBS and centrifuged at 16000 g for 30 min at 4 °C. The ApoVs were then obtained for further assay.

The morphology of the ApoVs was observed by transmission electron microscope (TEM) (JEM-1400FLASH, JEOL, Japan). Annexin V-FITC Apoptosis Detection Kit (BMS500FI-100, Thermo Fisher Scientific, USA) was applied to detect the apoptotic SHED. Western blotting was performed to characterize the specific protein expression in ApoVs. For NTA analysis, ApoVs were diluted in PBS and measured using a nanoparticle tracking analyzer (Particle Metrix, ZetaVIEW, Germany).

For in vivo experiments, ApoVs were resuspended in PBS at 20 μg/mL and injected into mice ear, while in vitro experiments, ApoVs were co-cultured with CD8^+^ T cells at a concentration of 10 μg/mL.

**Flow Cytometry Analysis.**

A single-cell suspension was prepared following the manufacturer’s instructions. 1×10^6^ isolated cells were stained with fluorophore-labeled antibodies: CD8-APC (MCD0827, R＆D system, USA, 0.2 µg/10^6^ cells), CD3-FITC (11-0031-85, eBioscience, USA,0.5 µg/test), CD90-PE (A15794, eBioscience, USA, 10 µL/1x10^6^ cells), CD105-PE (MA1-80944, eBioscience, USA, 10 µL/1x10^6^ cells), CD73-PE (12-0739-42,eBioscience, USA, 5 µL/test), CD29-PE (12-0291-81, eBioscience, USA, 1 µg/test), CD14-PE (ab307635, abcam, UK, 5 µL/10^6^ cells), HLA-DR-PE (ab64676, abcam, UK, 5 µL/10^6^ cells), CD45-FITC (RMCD45RC01, eBioscience, USA, 0.2 µg/10^6^ cells), CD34-PE (CD34-581-04, eBioscience, USA, 5 µL/10^6^ cells), CD79a-FITC (ab275943, abcam, UK, 5 µL/10^6^ cells).

**Quantitative Real-Time PCR.**

Ear tissues were collected and grinded liquid nitrogen freezing. Then the total RNA was extracted with TRIzol (Life Technologies, United States) and measured with a Nanodrop 2000 UV–visible spectrophotometer. Total RNA ranging from 20 to 100 ng/mL was utilized to create cDNA through a high capacity of cDNA reverse transcription kit (Analytik Jena, USA), following the provided guidelines. SYBR Green probes specific for Inf-γ, Il-1𝛽, Il-6, Cxcl9, Cxcl10, and Cxcl11 were used in quantitative real-time PCR analysis of cDNA. Information regarding the primers utilized is available in Table S1 (Supporting Information). The C1000 Touch PCR System (Bio-Rad, United States) was used for the analysis.

The relative gene expressions of (Ifn-γ, Il-1𝛽 , Il-6, Cxcl9, Cxcl10, and Cxcl11)were tested by qRT-PCR. The primers used are listed in below:

Table S1. qRT-PCR Primer Sequences

| Genes | Primer sequence (5′–3′)  (forward/reverse) | Product size (bp) |
| --- | --- | --- |
| *Ifn-γ* | ATGAACGCTACACACTGCATC | 182 |
|  | CCATCCTTTTGCCAGTTCCTC |  |
| *Il-1𝛽* | GCAACTGTTCCTGAACTCAACT | 89 |
|  | ATCTTTTGGGGTCCGTCAACT |  |
| *Il-6* | TAGTCCTTCCTACCCCAATTTCC | 76 |
|  | TTGGTCCTTAGCCACTCCTTC |  |
| *Cxcl9* | TCCTTTTGGGCATCATCTTCC | 110 |
|  | TTTGTAGTGGATCGTGCCTCG |  |
| *Cxcl10* | CCAAGTGCTGCCGTCATTTTC | 157 |
|  | GGCTCGCAGGGATGATTTCAA |  |
| *Cxcl11* | GGCTTCCTTATGTTCAAACAGGG | 108 |
|  | GCCGTTACTCGGGTAAATTACA |  |
| Gapdh | AGGTCGGTGTGAACGGATTTG | 123 |
|  | TGTAGACCATGTAGTTGAGGTCA |  |

**Terminal Deoxynucleotidyl Transferase dUTP Nick End Labeling (TUNEL) and CD8^+^ T Cells Staining**

To verify the apoptosis of CD8^+^ T cells after ApoVs treatment, ear samples were collected 6 h after ApoVs injection. The sections were stained utilizing a one-step TUNEL in situ apoptosis kit (E-CK-A322, Elabscience) following the manufacturer's protocol. Consequently, the apoptotic cells were visualized in red fluorescence. Subsequently, the mouse CD8 alpha Alexa Fluor 488 Mab (FAB116G-100, R＆D Systems, USA, diluted 1:200) was applied and incubated at 37°C for 1 h to directly label CD8^+^ T cells with green fluorescence. Sections were sealed with a mounting medium containing 4,’-6-diamidino-2-phenylindole (DAPI; ab104139, Abcam, UK) after washed with PBS for 3 times.

**SHED-ApoVs Co-culture with CD8^+^ T cells**

Each confocal dish was coated with Poly-D-Lysine at 37°C for 30 min to enhance their adhesive capacity for CD8^+^ T cells. Subsequently, CD8^+^ T cells (1x10^6^) pre-labeled with PKH67 (MINI67, Sigma, Germany) (as previously mentioned) were seeded onto the confocal dishes and cultured for 6 h to allow them to adhere to the dish surface. Then, SHED-ApoVs pre-labeled with PKH26 (MIDI26, Sigma, Germany) were added, and at time points of 0.5 h, 1 h, 1.5 h, 2 h, 2.5 h, 3 h, 3.5 h, 4 h, 4.5 h, 5 h, 5.5 h, 6 h, 6.5 h and 7 h post-addition, the supernatant was aspirated and the cells were fixed with paraformaldehyde. Furthermore, the cell nucleus was stained with 4,’-6-diamidino-2-phenylindole (DAPI; ab104139, Abcam, UK). Finally, the vesicle-cell membrane interactions, including contact and fusion, were observed and photographed using a spinning disk confocal super resolution microscope (IXplore SpinSR, Olympus, Japan) at specific time points.

**REFERENCES**

[1] K. Xuan, B. Li, H. Guo, W. Sun, X. Kou, X. He, Y. Zhang, J. Sun, A. Liu, L. Liao, S. Liu, W. Liu, C. Hu, S. Shi, Y. Jin, *Sci Transl Med* **2018**, *10* (455), eaaf3227

[2] Z. Li, M. Wu, S. Liu, X. Liu, Y. Huan, Q. Ye, X. Yang, H. Guo, A. Liu, X. Huang, X. Yang, F. Ding, H. Xu, J. Zhou, P. Liu, S. Liu, Y. Jin, K. Xuan, *Mol Ther* **2022**, *30* (10), 3193.

**Supplementary Figure Legends**

**
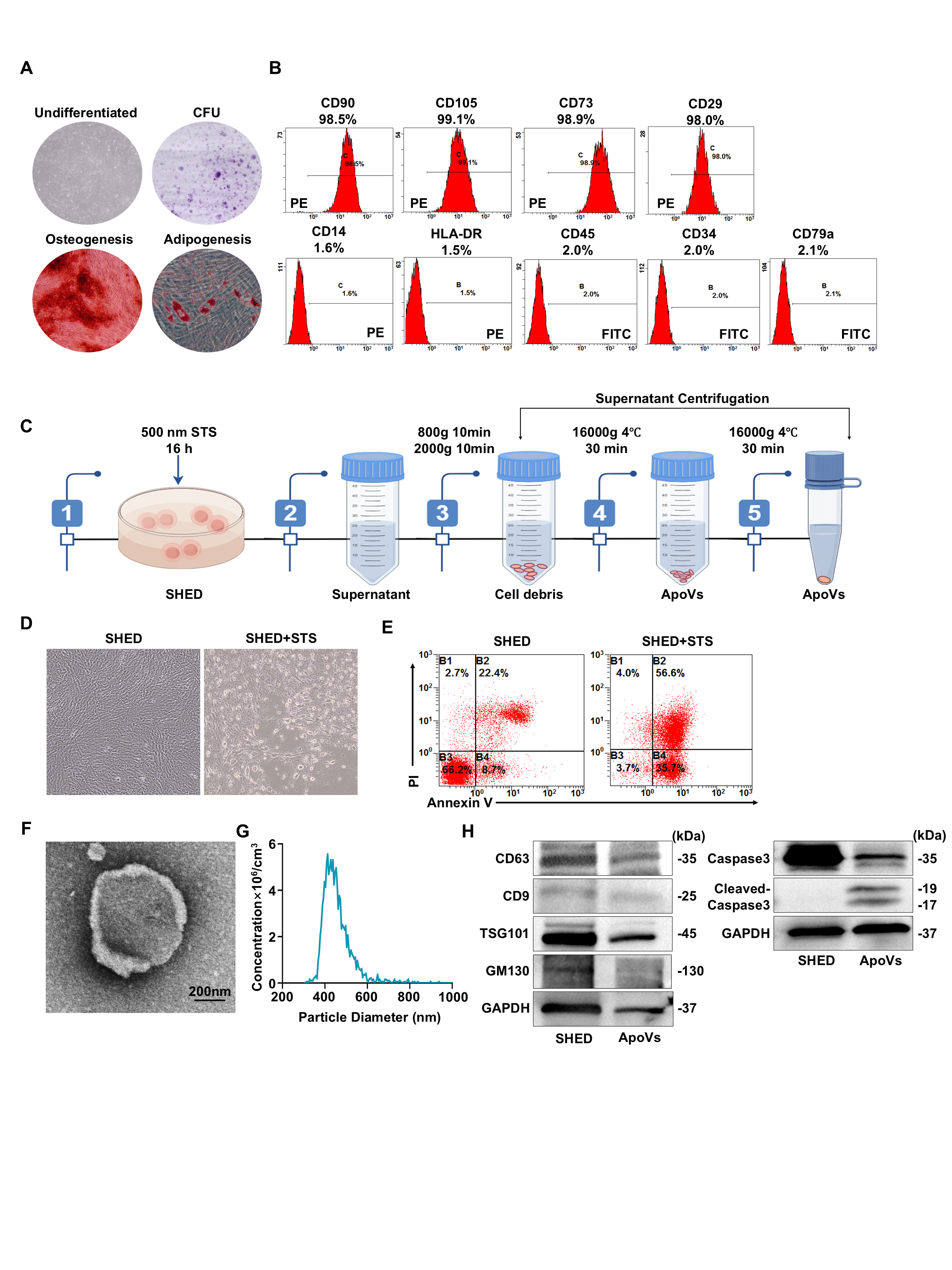
**

**Figure S1. Isolation and Characterization of MSCs and MSCs-ApoVs.**

A)Self-renewal and multipotent differentiation capacity of SHED. CFU: Colony-Forming Unit. B) Postive expression of MSCs surface molecules (CD90, CD105, CD73, CD29) and negtive expression of leukocyte and hematopoietic progenitor cell surface markers (CD14, HLA-DR, CD45, CD34 and CD79a). C) Schemes of SHED-ApoVs isolation. D) The characteristic morphology changes in apoptosis of SHED were obvious by microscope. E) Flow cytometry analysis of Annexin V/PI double staining shows the elevated percent of apoptosis SHED. F) Transmission electron microscopy (TEM) analysis of ApoVs structure. G) Nanoparticle tracking analysis (NTA) analysis of ApoVs. H) Western bolt analysis of CD63, CD9, TSG101, GM130, caspase 3 and cleaved-caspase 3 in SHED and SHED-ApoVs.

**
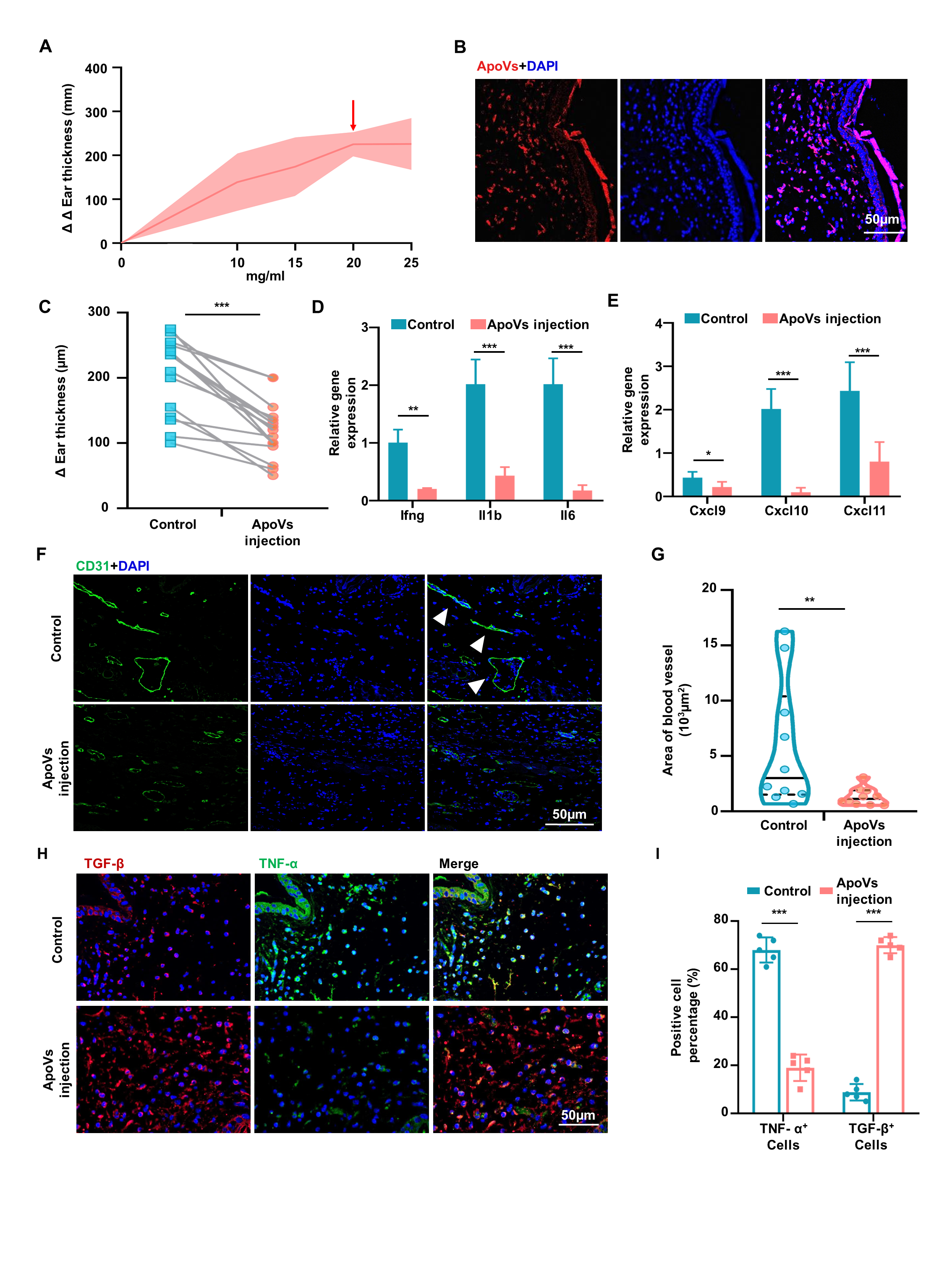
**

**Figure S2. ApoVs-afforded Anti-hypersensitivity is Associated with CD8^+^ T Cells Dysfunction.**

A) The thickness difference between the left and right ears in different concentrations of ApoVs (n=5). B) The distribution of PKH26-labeled ApoVs at the site of ear lesions was analyzed by immunofluorescence staining. Scale bar: 50 μm. C) Ear thickness was measured 24 h after the elicitation. The increase in ear thickness from the baseline was shown (n=15). D) qRT-PCR showed the ear tissue gene expression of Ifng (IFN-γ), Il1b (IL-1β) and Il6 (IL-6) decreased after ApoVs injection (n=3). E) qRT-PCR showed the ear tissue gene expression of Cxcl9, Cxcl10 and Cxcl11 decreased after ApoVs injection (n=3). F) Immunostaining of ear lesions for CD31 (green) and DAPI (blue). Scale bar: 50 μm. G) Quantification of the dermal blood vascular area per section (n=10). H) Representative TGF-β (red) and TNF-α-positive (green) cells in the tissue sections of control mice or ApoVs-treated mice. Scale bar: 50 μm. I) The percentage of TGF-β and TNF-α-positive cells in each group.

**
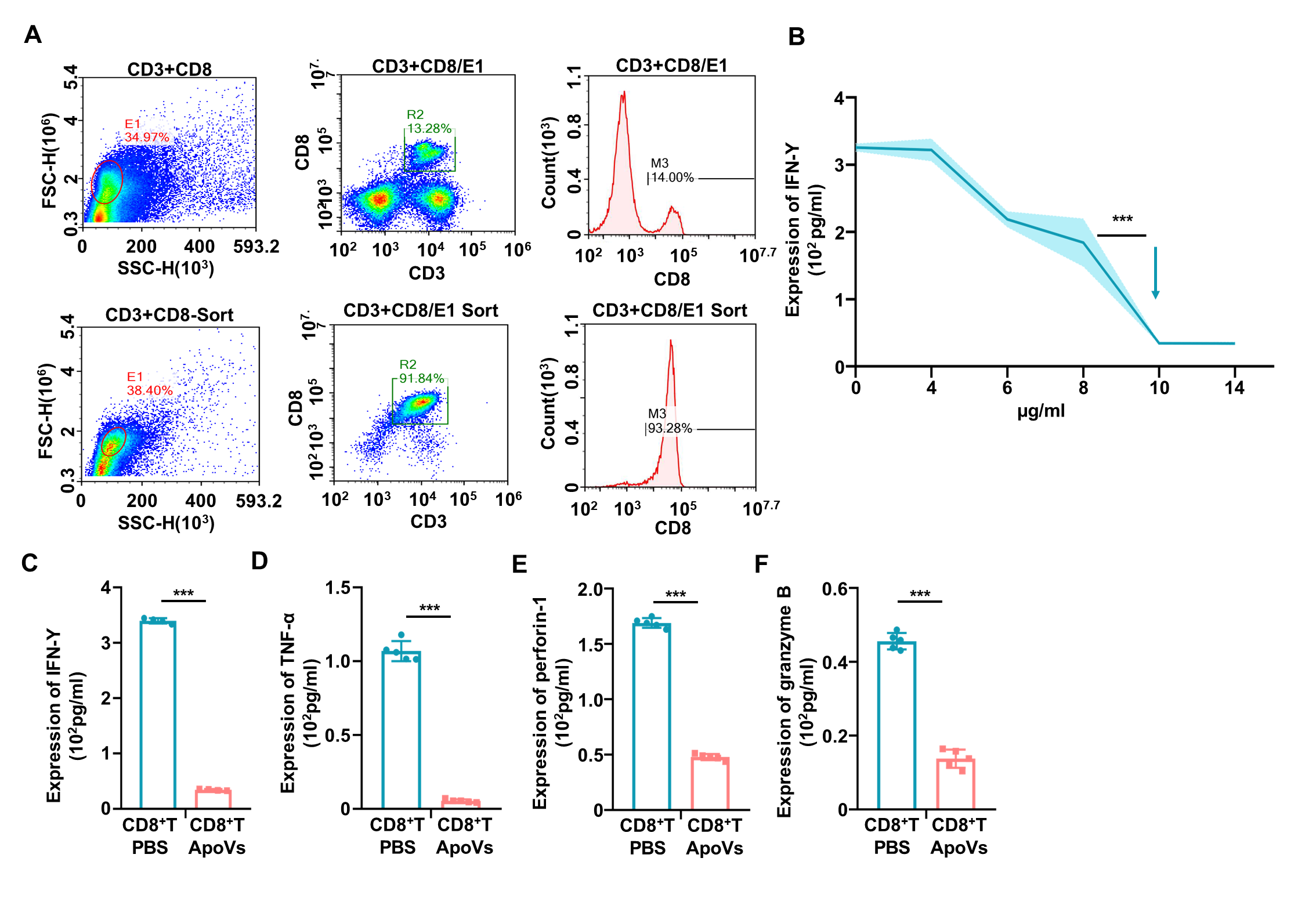
Figure S3. ApoVs Treatment Attenuated function of CD8^+^ T cells *in vitro*.**

A) Flow cytometry showed the sorted CD8^+^ T cell positive express CD3 and CD8. B) ELISA showed the IFN-γ production level in different concentrations of ApoVs in vitro (n=5). C-F) ELISA displayed the expression of CD8^+^ T cell cytotoxic function cytokines including IFN-γ, TNF-α, RPF1 and GZMB all decreased after 10 μg/mL ApoVs applied in CD8^+^ T cells (n=5). ** *P* < 0.01, ****P* < 0.001.

**
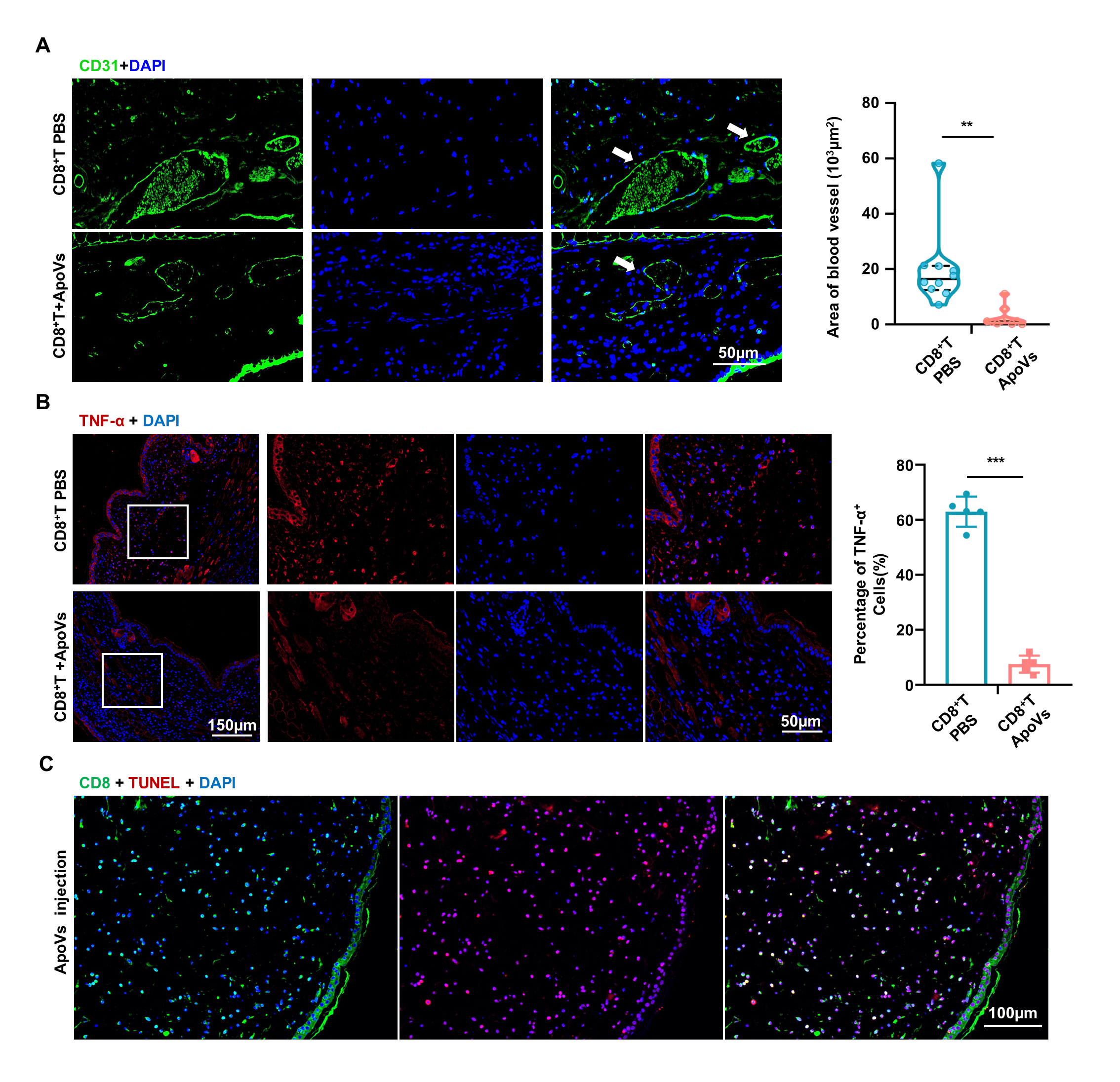
**

**Figure S4. ApoVs Treatment Attenuated CD8^+^ T cells Mediated Ear Hypersensitivity in Adoptive Transfer Mice.**

A) Immunostaining of ear lesions for CD31 (green) and DAPI (blue). Scale bar: 50 μm. Quantification of the dermal blood vascular area per section (n=10). B) Representative TNF-α-positive cells (red) in ear tissue. Scale bar: 150 μm. Boxed scale bar: 50 μm. The percentage of TNF-α-positive cells in each group (n=5). C) Co-localization of TUNEL positive cells (red) and CD8^+^ T cells (green). Scale bar: 100 μm. ** *P* < 0.01, ****P* < 0.001.


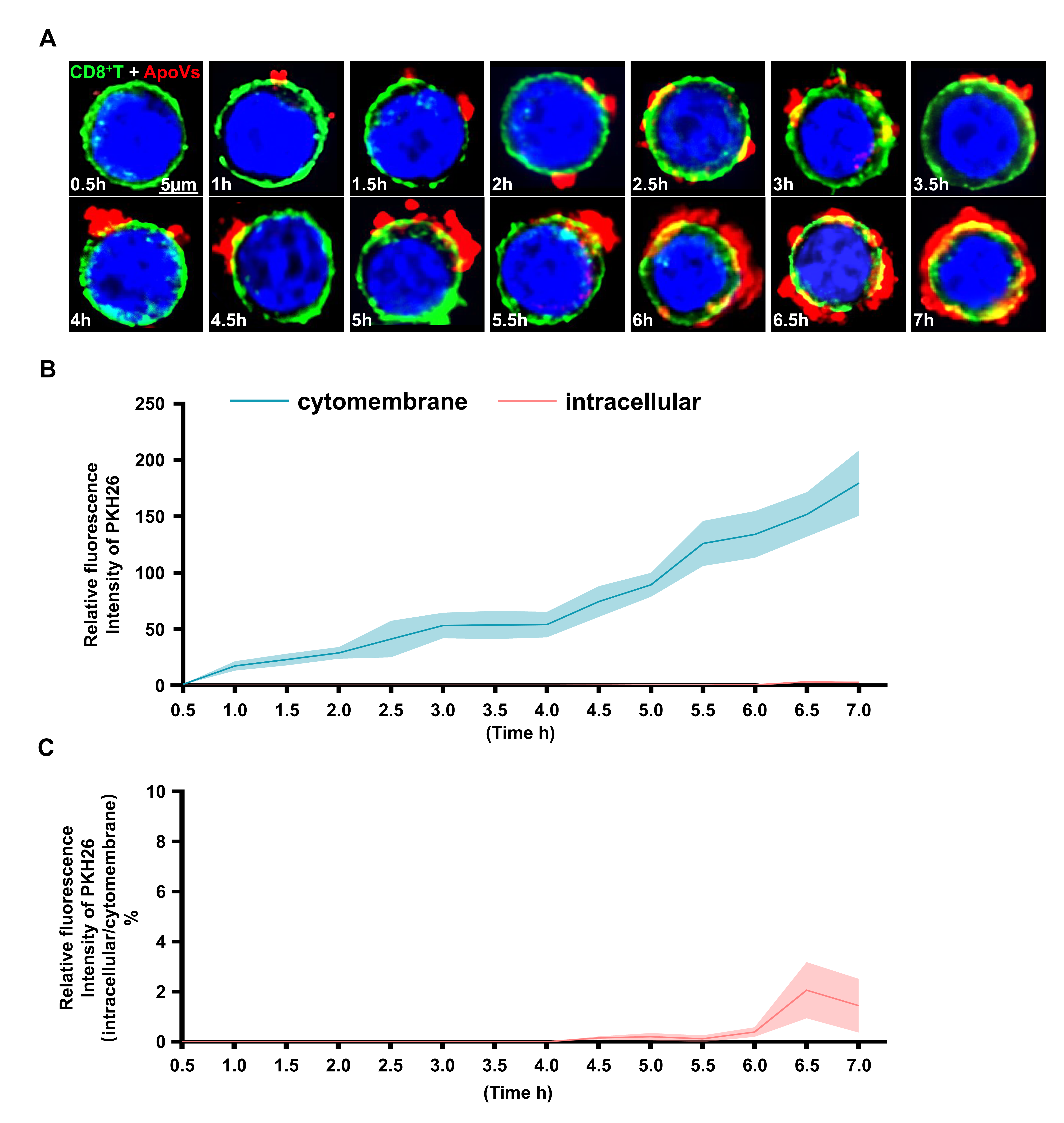


**Figure S5. ApoVs Interact with CD8^+^ T Cells Mainly through Membrane Fusion.**

A) ApoVs uptake experiment showed the ApoVs (PKH26, red) interact with CD8^+^ T cells (PKH67, green) at specific time points. Cell nucleus: blue. Scale bar: 5 μm. B) Quantification of intracellular and cytomembrane PKH26 (ApoVs) fluorescence intensity. C) Quantification of the the ratio of intracellular and cytomembrane fluorescence intensity of PKH26 (ApoVs).

**
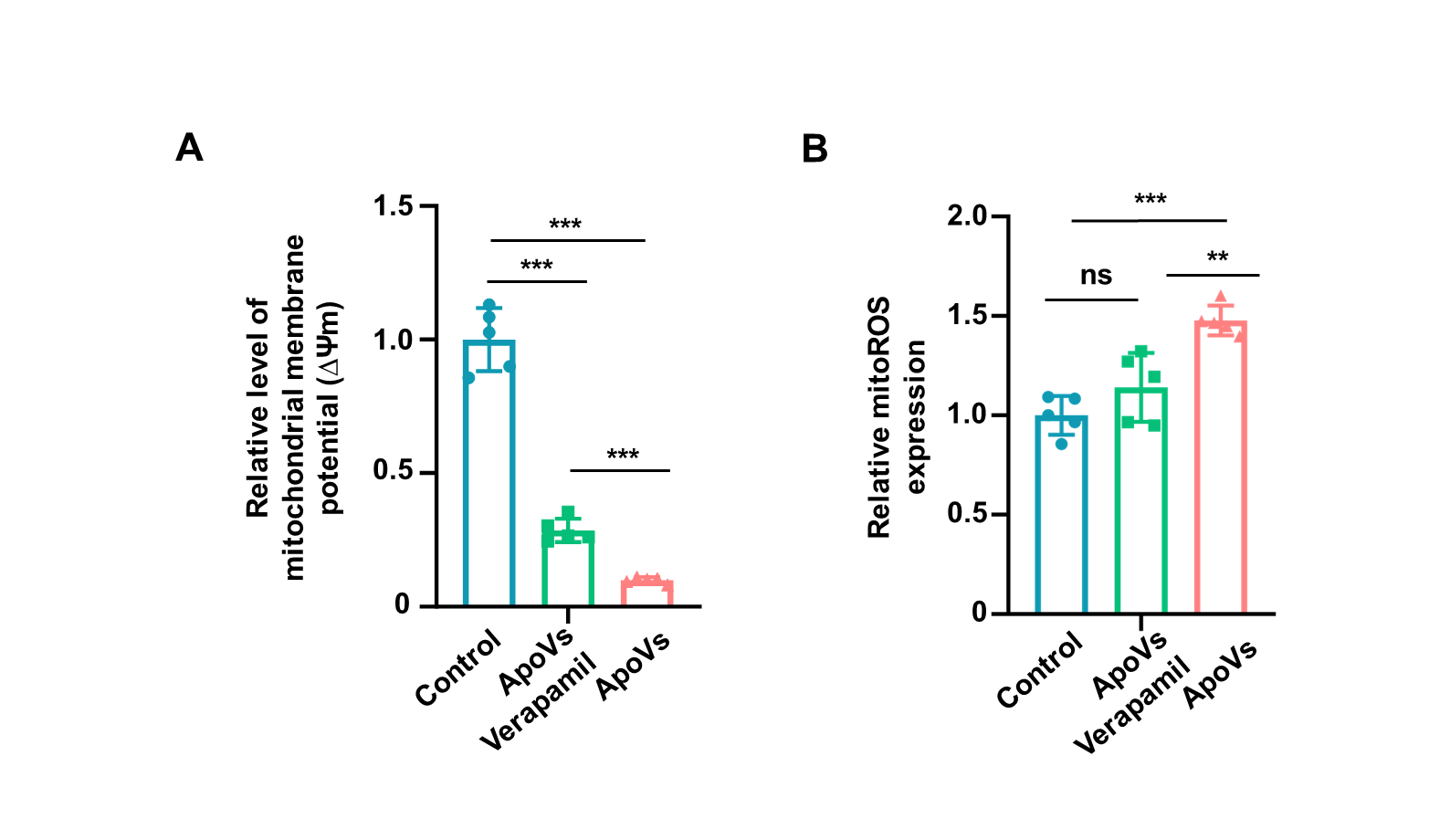
**

**Figure S6. Inhibiting Calcium Influx Alleviated Mitochondrial Function.**

A) Mitochondrial membrane potential (∆Ψm) were analyzed by the relative ration of JC-1 aggregates (OD=525) and monomer (OD=490) (n=5). J) Relative mitochondrial ROS level of three groups (n=5). ** *P* < 0.01, ****P* < 0.001.


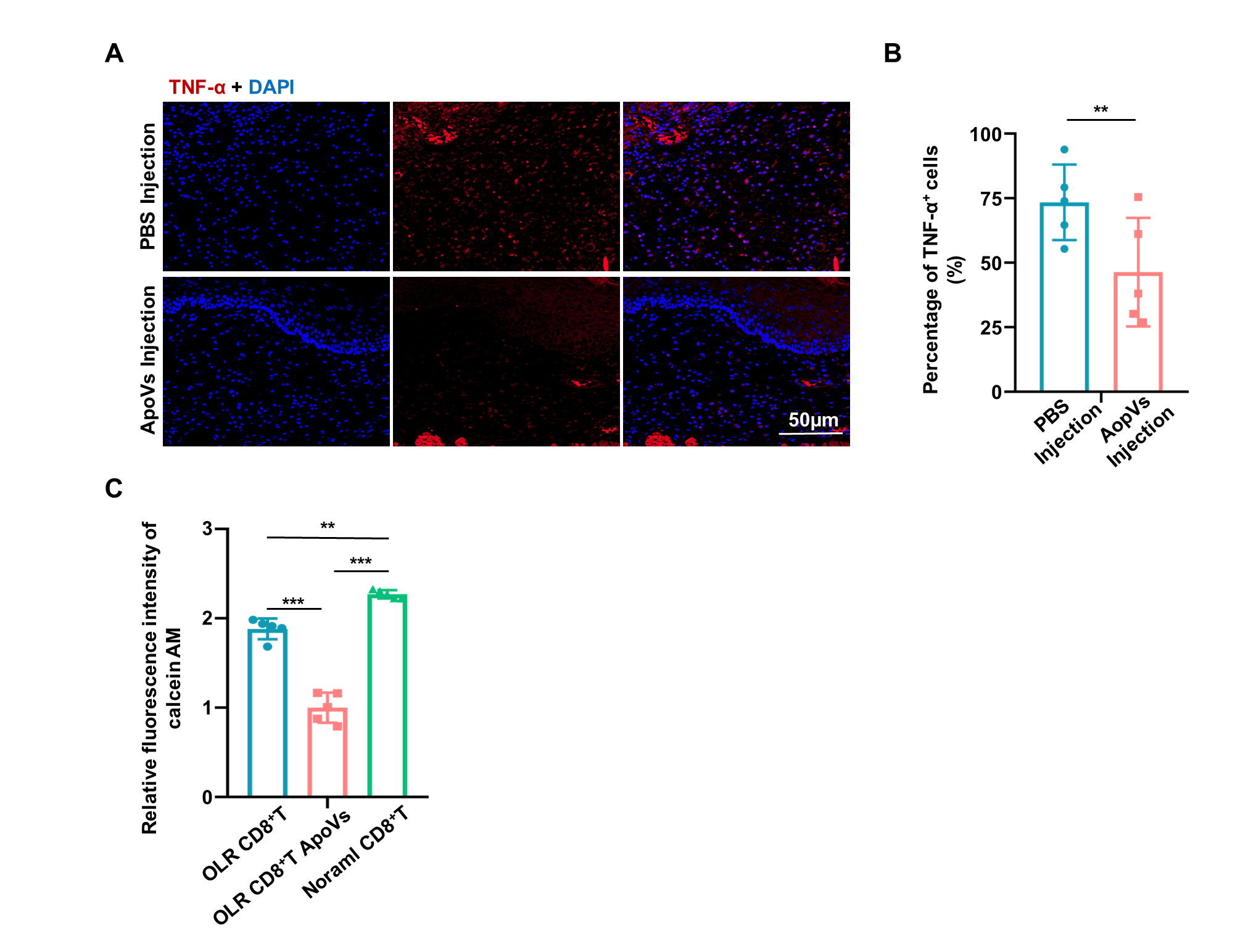


**Figure S7. ApoVs Treatment Alleviated Oral Lichen Reactions.**

A) Representative TNF-α-positive cells (red) in mucosal tissue (n=5). Scale bar: 50 μm. B) The percentage of TNF-α-positive cells in each group. C) Quantification of relative fluorescence intensity of Calcein AM in three groups (n=5). ** *P* < 0.01, ****P* < 0.001.
